# Design and estimation in clinical trials with subpopulation selection

**DOI:** 10.1002/sim.7925

**Published:** 2018-08-07

**Authors:** Yi‐Da Chiu, Franz Koenig, Martin Posch, Thomas Jaki

**Affiliations:** ^1^ Medical and Pharmaceutical Statistics Research Unit, Department of Mathematics and Statistics Lancaster University Lancashire UK; ^2^ Center for Medical Statistics, Informatics, and Intelligent Systems Medical University of Vienna Vienna Austria

**Keywords:** bias, enrichment design, maximum likelihood estimator, prevalence, subgroup analysis, subpopulation selection

## Abstract

Population heterogeneity is frequently observed among patients' treatment responses in clinical trials because of various factors such as clinical background, environmental, and genetic factors. Different subpopulations defined by those baseline factors can lead to differences in the benefit or safety profile of a therapeutic intervention. Ignoring heterogeneity between subpopulations can substantially impact on medical practice. One approach to address heterogeneity necessitates designs and analysis of clinical trials with subpopulation selection. Several types of designs have been proposed for different circumstances. In this work, we discuss a class of designs that allow selection of a predefined subgroup. Using the selection based on the maximum test statistics as the worst‐case scenario, we then investigate the precision and accuracy of the maximum likelihood estimator at the end of the study via simulations. We find that the required sample size is chiefly determined by the subgroup prevalence and show in simulations that the maximum likelihood estimator for these designs can be substantially biased.

## 1 INTRODUCTION

Heterogeneity is frequently observed among patients' treatment response in clinical trials. This is due to various factors such as age, race, disease severity, or genetic differences. The topic of heterogeneity in treatment effects has received some attention in the literature (eg, see related works^1^, ^2^, ^3^) and graphical methods such as forest plots are routinely used for the purpose of examining heterogeneity in effects (eg, the work of Cuzick^4^). Ignoring heterogeneity can substantially impact on medical practice. For example, a treatment might work well in some patients but not in others. Naively estimating the treatment effect across all patients will result in a diluted effect for the group that truly benefits from the treatment. At the same time, an ethical issue arises due to delivering a treatment to all patients, whereas some might not expect an effect and will potentially be exposed to harmful side effects. To address these issues, trials that consider (potential) subgroups defined by one or more biomarkers are becoming more popular. In general, a biomarker is some measurable variable that might help to identify distinct groups of patients and some examples include cholesterol levels, genetic variations, or age. A biomarker is considered prognostic if it provides information about the value of some other variable of interest (eg, the primary endpoint of a study), whereas it is called predictive if its value yields information about the treatment effect. In this paper, we will only consider the latter type of biomarkers.

A number of different designs concerning treatment selection and subgroups within the study populations have been proposed. These designs can be categorized by factors such as design setting (confirmatory or exploratory) or methodology (frequestist, Bayesian, or utility/decision function) (see related works^5^, ^6^, ^7^). Additionally, the designs can be categorized into single‐stage (fixed sample) designs and multistage (adaptive) designs. Both conventionally utilize multiple testing procedures to test for effects in each of the populations of interest. An overview of different multiple testing approaches for this purpose is given in the work of Alosh et al^6^ and the references therein. A single‐stage design with one biomarker tests, for example, the null hypotheses, ie, the treatment effect of the full population is zero, ie, H_0F_ and the treatment effect in the subgroup of interest is zero, ie, H_0S_.^5^, ^8^, ^9^, ^10^, ^11^, ^12^ These designs are usually employed for exploratory subgroup analysis in phase II (ie, to identify an interesting subgroup) or for confirmatory subgroup analysis in phase III, examining the treatment benefit of prespecified subgroups. Corresponding multistage designs are constructed either as extensions of group sequential approaches^13^ or using combination tests.^14^ They can refine the population to either the whole or one or more subgroups at the interim analysis and can allow for early stopping for benefit and lack of benefit (see, eg, other works^5^, ^15^, ^16^, ^17^, ^18^).

The accuracy and precision of the treatment effect estimators in subgroup analysis are also crucial to the development of novel treatments and decisions about treatment implementation. Especially, bias is ubiquitous in designs that select (see the work of Bauer et al^19^) and, in the designs considered here, the bias can come from selecting which (sub)population should be studied further or from selective reporting promising results even in a simple fixed sample design. A variety of papers on treatment effect estimation in the related problem of trials with treatment selection have been published. Approximate bias‐correction estimators for single‐stage designs for normal endpoints are discussed in the works of Shen^20^ and Stallard et al,^21^ uniformly minimum variance conditional unbiased estimators for two stage designs have been proposed by Cohen and Sackrowitz,^22^ and further extensions are published in the works of Bowden and Glimm^23^ Sill and Sampson.^24^ Shrinkage estimators have been discussed in the work of Carreras and Brannath,^25^ whereas approaches to construct confidence intervals are described in related works.^26^, ^27^, ^28^ Time‐to‐event endpoints are considered in the work of Brückner et al.^29^

In contrast, rather limited literature addresses estimation issues in clinical trials with subpopulation selection. For single‐stage designs, Rosenkranz^30^ proposed a bias‐adjustment method employing bootstrap techniques to calibrate the estimates upon general distributional assumption on outcomes. For multistage designs, Kimani et al^31^ proposed two estimators, ie, one is a naive estimator using a weighted average of per‐stage means and prevalences for each subgroup and the other is a uniformly minimum variance conditional unbiased estimator derived by the Rao‐Blackwell theorem. They assessed the performance under several situations, such as different values of prevalence and treatment effect of one subpopulation, and also suggested which estimator should be used according to what population is selected at Stage 1. In addition, Magnusson and Turnbull^16^ focused on the designs rather than estimation, though they outlined an extended bias‐reduction algorithm proposed by Wang and Leung^32^ in which uses double bootstrap methods^33^ to adjust ML‐estimates and build bootstrap confidence interval.

Despite some contributions on estimation, the aforementioned papers do not provide a complete overview of the maximum likelihood estimator (MLE) under various designs and lack exploring the estimator performance in further conditions. Rosenkranz's^30^ simulation work on single‐stage designs implicitly regarded the MLE only in circumstances with few different treatment effects for subgroups and thresholds used in the selection rule. Kimani et al^31^ considered two‐stage adaptive seamless designs, selecting subpopulation based on the Stage 1 data but not allowing early stopping, and they only assessed estimators with selection but without reporting promising results. The multistage designs of Magusson and Turnbull allow to select multiple subpopulations if the estimates of treatment effects are above certain thresholds at Stage 1.

In this paper, we discuss a framework to design single and multistage design that select subgroups. We illustrate the design properties when selection is based on the maximum statistic and comprehensively evaluate the properties of the MLE for these designs. Note that selecting on the basis of the maximum statistic is the worst case for both type I error (provided that the number of hypothesis remains the same) and bias and hence of particular interest. In Section 2, we derive a subgroup selection design that selects groups based on the maximum test statistic. Section 3 describes a simulation study in which different general design scenarios are evaluated and the bias and MSE of the corresponding MLEs are derived. In Section 4, we remark on the designs with different selection rules, then summarize the results of the simulation study and discuss its implications for future work.

## 2 DESIGNS

In this section, we first define the basic setting and notation and then provide general ideas for designs with subpopulation selection based on the maximum test statistic.

### 2.1 Basic setting and notation

Assume J mutually disjoint subpopulations are in the full study population (F ) and denote the prevalence of the jth subpopulation (S_j_) by λ_j_, where j = 1,…,J and $\sum \lambda_{j}=1$. The sample size of each subgroup is fixed as a proportion of the total sample size depending on the respective prevalence. We use n_j_ to denote the sample size in subgroup S_j_ and more generally use subscripts to denote groups and treatments and superscripts for stages. We consider a normally distributed endpoint with mean μ_j,l_ with j = 1,…J and l = T,C, where subscript T corresponds to the treatment group and C to the control group. Additionally, we assume a common variance, ie, σ ^2^, across subpopulations.

#### 2.1.1 Single‐stage design

For a single‐stage design, the test statistics used for selection and decision are distributed as

$$Z_{j}^{\left(1\right)}=I_{j}^{\left(1\right)}\left({\bar{Y}}_{j,T}^{\left(1\right)}-{\bar{Y}}_{j,C}^{\left(1\right)}\right)∼N\left(I_{j}^{\left(1\right)}\theta_{j},1\right).$$

Note that we use the (unnecessary) superscript (1) for consistency with the multistage notation used later. ${\bar{Y}}_{j,T}^{\left(1\right)}$ and ${\bar{Y}}_{j,C}^{\left(1\right)}$ are the sample means of the treatment group and of the control group within S_j_, respectively. The true treatment difference in S_j_ is denoted as θ_j_ = μ_j,T_ − μ_j,C_ and $I_{j}^{\left(1\right)}=1/(\sigma \sqrt{1/n_{j,T}^{\left(1\right)}+1/n_{j,C}^{\left(1\right)}})$ is the information level for S_j_. This further simplifies to $1/(2\sigma \sqrt{1/n_{j}^{\left(1\right)}})$ when the assumed treatment allocation ratio is 1:1, where $n_{j}^{\left(1\right)}$ is the total sample size of S_j_ until the end of Stage 1.

Considering a composite population $S_{U+V}$, combining two subpopulations $S_{U}$ and $S_{V}$ (where U,V ⊆{1,2,…,J}, U∩V=∅ ), the test statistics are distributed as

$$Z_{U+V}^{\left(1\right)}=\sqrt{\frac{n_{U}^{\left(1\right)}}{n_{U+V}^{\left(1\right)}}}Z_{U}^{\left(1\right)}+\sqrt{\frac{n_{V}^{\left(1\right)}}{n_{U+V}^{\left(1\right)}}}Z_{V}^{\left(1\right)}=I_{{}_{U+V}}^{\left(1\right)}\left({\bar{Y}}_{{\;}_{U+V},T}^{\left(1\right)}-{\bar{Y}}_{{\;}_{U+V},C}^{\left(1\right)}\right)∼N\left(I_{{}_{U+V}}^{\left(1\right)}\left(\mu_{{}_{U+V},T}-\mu_{{}_{U+V},C}\right)\;,1\right),$$

where ${\bar{Y}}_{{\;}_{U+V},T}^{\left(1\right)}$ and ${\bar{Y}}_{{\;}_{U+V},C}^{\left(1\right)}$ are defined as before but the observations are from the combined treatment group and the combined control group of the united subpopulation $S_{{}_{U+V}}$. The true treatment effect size and the information level of $S_{{}_{U+V}}$ are $\theta_{{}_{U+V}}=\mu_{{}_{U+V},T}-\mu_{{}_{U+V},C}$ and $I_{{}_{U+V}}^{\left(1\right)}=1/(\sigma \sqrt{1/n_{{}_{U+V},T}^{\left(1\right)}+1/n_{{}_{U+V},C}^{\left(1\right)}})$, respectively. $I_{{}_{U+V}}^{\left(1\right)}$ is also equal to $1/(2\sigma \sqrt{1/(n_{{}_{U}}^{\left(1\right)}+n_{{}_{V}}^{\left(1\right)})})$ for equal allocation. Additionally, $\theta_{{}_{U+V}}=(\lambda_{{}_{U}}\theta_{{}_{U}}+\lambda_{{}_{V}}\theta_{{}_{V}})/(\lambda_{{}_{U}}+\lambda_{{}_{V}})$. Note that, if U and V are complementary, their composite population $S_{{}_{U+V}}$ is the full population F and then the subscript of the aforementioned notations are replaced with f. If U and V have an individual element for each, such as {1} and {2}, we simplify the notation of U+V as 1 + 2. This notation simply denotes the union of $S_{{}_{U}}$ and $S_{{}_{V}}$, and it does not necessarily imply that one is nested in the other.

#### 2.1.2 Multistage design

For multistage designs, the test statistic based on the accumulated data at the end of stage k (k ≤ K, the total stage number) for $S_{{}_{U}}$ is denoted by

$$Z_{{}_{U}}^{1:k}=\sum_{i=1}^{k}\sqrt{\frac{I_{{}_{U}}^{\left(i\right)}}{I_{{}_{U}}^{1:k}}}Z_{{}_{U}}^{\left(i\right)}=I_{{}_{U}}^{1:k}\left({\bar{Y}}_{{\;}_{U},T}^{1:k}-{\bar{Y}}_{{\;}_{U},C}^{1:k}\right)∼N\left(I_{{}_{U}}^{1:k}\theta_{{\;}_{U}},1\right),$$

where the superscript 1:k refers to a quantity calculated based on the accumulated data at the end of stage k; therefore, $I_{{}_{U}}^{1:k}$ is the accumulated information level defined accordingly as $1/(\sigma \sqrt{1/n_{{}_{U},T}^{1:k}+1/n_{{}_{U},C}^{1:k}})$.

### 2.2 Designs considered

We consider designs that control the family‐wise error rate (FWER) at level α in the strong sense^34^ and the set of hypotheses to be tested

$$H_{0s}:\theta_{s}\leq 0\;\text{versus}\;H_{as}:\theta_{s}>0,\;s\in S,$$

where S is the index set corresponding to the subpopulations considered and can index nested groups. For instance, if we consider subgroup 1, subgroup 1 and 2, or the full population being of interest, S={1,1+2,f}.

#### 2.2.1 Single‐stage designs

To select, we use the maximum of the test statistics among $Z_{s}^{\left(1\right)}$, s∈S for population selection. Its implication and other selection rules will be discussed later. In the evaluation of the operating characteristics, we consider the case where population selection is undertaken first and only subsequently the corresponding hypothesis being tested. The testing procedure is making a decision about rejecting H_0w_ if $Z_{w}^{\left(1\right)}\geq C_{\alpha }$, where w is a realized value of the random variable W and refers to the event that subpopulation S_w_ is chosen. $Z_{w}^{\left(1\right)}$ is the selected test statistic for S_w_, and C_α_ is the corresponding critical value found to ensure the FWER in the strong sense.

The crucial element to find the appropriate critical value and sample size is the density of the joint distribution of the selected test statistic $Z_{W}^{\left(1\right)}$ and the selected population index W. While the subsequent results are derived on the basis of selecting based on the maximum statistic, other selection rules can equally be implemented. Using a different rule results in a different density and, for illustration purposes, we also provide the resulting distribution for selecting any populations whose estimated effect exceeds a prespecified value, ie, δ, in the Supplementary Materials S.6. The joint densities $p_{Z_{W}^{\left(1\right)},W}(z_{w}^{\left(1\right)},w;\Theta )$, w∈S govern the probability whether to select S_w_ and to reject the null hypothesis H_0w_ (where **Θ** is a configuration of all mutually disjoint subgroup treatment effects θ_1_,θ_2_,…,θ_J_). It can further be decomposed as $p_{Z_{w}^{\left(1\right)}}(z_{w}^{\left(1\right)};\Theta )\cdot \text{Pr}(W=w|Z_{w}^{\left(1\right)}=z_{w}^{\left(1\right)};\Theta )$. Consequently, the joint densities of $Z_{W}^{\left(1\right)}$ and W can be represented as

(1) $$p_{Z_{W}^{\left(1\right)},W}\left(z_{w}^{\left(1\right)},w;\Theta \right)=\varphi \left(z_{w}^{\left(1\right)}-\theta_{w}I_{w}^{\left(1\right)}\right)\Psi_{S\setminus w}\left(z_{w}^{\left(1\right)},\text{⋯},z_{w}^{\left(1\right)};\Theta \right),$$

where φ denotes the standard normal density; and $\Psi_{S\setminus w}(\cdot ,\text{⋯},\cdot ;\Theta )$ is the cumulative distribution function of the |S|−1‐dimensional normal distribution conditional on $Z_{w}^{\left(1\right)}$ under a specified configuration of treatment effects **Θ**, where |S| is the cardinality of S. The covariance matrix depends on whether subgroups are nested or not (see examples in Supplementary Materials S.2 and S.3). The cumulative distribution function specifies $\text{Pr}(W=w|Z_{w}^{\left(1\right)}=z_{w};\Theta )$. It is noted that (1) is similar to the integrand of equation (4) in the work of Spiessens and Debois,^9^ where two coprimary analyses are performed on the full population and a subgroup, and the significance level for F is prespecified.

Using an iterative search, C_α_ can then be found using the following inequality:

(2) $$\alpha \geq \sum_{w\in S}\int_{C_{\alpha }}^{\infty }p_{Z_{W}^{\left(1\right)},W}\left(z_{w}^{\left(1\right)},w;\Theta_{0}\right)dz_{w}^{\left(1\right)},$$

where **Θ**=**Θ**_0_ denotes the global null hypothesis H_0_, θ_1_ = θ_2_ = … = θ_J_ = 0. Note that finding the critical value under this setting implies weak control of the FWER. Following the work of Magirr et al,^35^ it can be shown, however, that weak control implies strong control since θ_1_ = θ_2_ = … = θ_J_ = 0 maximizes the type I error when selection is based on the maximum. Similarly, assume an alternative hypothesis that exactly one subgroup (say S_w_, w in S) has nonzero positive effect size, ie, δ, but others have none is true, the required total sample size for the full population $n_{f}^{\left(1\right)}$ can be found using the aforementioned critical values, a desired effect, and a specified power level, ie, 1 − β. The related equation is

(3) $$1-\beta \leq \int_{C_{\alpha }}^{\infty }p_{Z_{W}^{\left(1\right)},W}\left(z_{w}^{\left(1\right)},w;\Theta_{a}\right)dz_{w}^{\left(1\right)},$$

where **Θ**_a_ denotes the alternative hypothesis, a vector of size J whose elements are all 0 except for the wth element, which is δ. The desired $n_{f}^{\left(1\right)}$ is obtained by iteratively increasing the sample size until Equation (3) holds.

**Figure 1:**
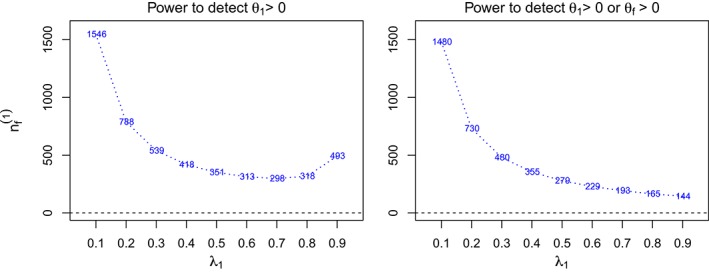
The total sample sizes of the full population F ( $n_{f}^{\left(1\right)}$) across prevalence rates of S_1_ (λ_1_) for two different definitions of power. The design is a single‐stage design with two subpopulations where the treatment effects θ_1_ and θ_2_ for S_1_ and S_2_ are 0.5 and 0, respectively. The type‐I error and power are specified at 0.025 and 80% [Colour figure can be viewed at http://wileyonlinelibrary.com]

Note that only rejection of the hypothesis with the truly largest effect is considered in this power requirement. Similar considerations can be used to find the power to reject any false null hypothesis (see Figure 1 for an example).

We have derived the aforementioned formula here for consistency, as for the multistage designs considered in the following, only the selected subgroup continues to subsequent stages.

The derivations of (2) and (3) are provided in the Supplementary Materials S.1 and more specific example solutions for the single‐stage design with two and three subgroups are given in Supplementary Materials S.2 and S.3 when the index set of selection population is S={1,f} and S={1,1+2,f}.

#### 2.2.2 Multistage designs

The multistage designs we consider follow similar procedures as the aforementioned single‐stage designs. Population selection is performed at the first interim analysis, but any population in S can be selected. We consider the case where data after Stage 1 are enriched so that the total sample size in the trial remains fixed but the sample size of subgroups that have not been selected is reallocated to the remaining populations. Suppose the selected population is *S*_*w*_, the difference is that, at stage *k*, the testing procedure stops by rejecting *H*_0*w*_ if $Z_{w}^{1:k}\geq C_{u_{k},\alpha }$, or stops with retaining *H*_0*w*_ if $Z_{w}^{1:k}\leq C_{l_{k}}$, or the procedure continues to stage *k* + 1 if $C_{l_{k}}\leq Z_{w}^{1:k}\leq C_{u_{k},\alpha }$, where $C_{u_{k},\alpha }$ and $C_{l_{k}}$ are the corresponding upper and lower stopping boundaries at stage *k*.

Two elements are required for appropriate stopping boundaries and stage‐wise sample sizes. The first is the joint density of $\left(Z_{W}^{\left(1\right)},W\right)$, as shown in (1). The second element is the density of the conditional distribution of the test statistics $Z_{w}^{1:k}$ (with accumulated data until stage *k*) given its precursor $Z_{w}^{1:(k-1)}$ at stage *k* − 1. We denote this conditional density by $p_{w,k|k-1}(z_{w}^{1:k}|z_{w}^{1:(k-1)};\Theta )$ and its general mathematical form is given in Supplementary materials S.4.

The stage‐wise density comprising of the two elements can then be used to determine the probability of stopping for efficacy or for futility at stage *k*. For example, the stage‐wise densities at Stage 2 with different values of *W* are specified as

(4) $$p_{Z_{W}^{\left(1\right)},W}\left(z_{w}^{\left(1\right)},w;\Theta \right)\cdot p_{w,2|1}\left(z_{w}^{1:2}|z_{w}^{1};\Theta \right),\;w\in S.$$

Then, given **Θ**=Θ_0_ (ie, under the global null hypothesis), the probability of early stopping at Stage 2 (either for lack of effect or early rejection) for the subgroup *S*_*w*_ can be calculated as

$$\int_{C_{l_{1}}}^{C_{u_{1},\alpha }}\int_{C_{u_{2},\alpha }}^{\infty }p_{Z_{W}^{\left(1\right)},W}\left(z_{w}^{\left(1\right)},w;\Theta_{0}\right)\cdot p_{w,2|1}\left(z_{w}^{1:2}|z_{w}^{\left(1\right)};\Theta_{0}\right)dz_{w}^{1:2}dz_{w}^{\left(1\right)},\;w\in S,$$

where the integral bounds signify that the design continues after Stage 1 but stops at Stage 2 for efficacy. The conditional function $p_{w,2|1}(z_{w}^{1:2}|z_{w}^{\left(1\right)};\Theta )$ is used to calculate stopping probability at Stage 2, given that the design does not stop at the preceding stage. Similarly, the stage‐wise densities at stage *k* are the product of the expression in (1), multiplying the factor $\prod_{m=k}^{1}p_{w,m|m-1}(z_{w}^{1:m}|z_{w}^{1:(m-1)};\Theta )$. The value of the *k*‐fold multiple integral within the integrand region defined by stopping boundaries before stage *k* + 1 is the early stopping probability at stage *k*. Each conditional density $p_{w,m|m-1}(z_{w}^{1:m}|z_{w}^{1:(m-1)};\Theta )$ with its respective integral bound controls the probability of whether the design stops or continues, given that the design has proceeded at the previous stage.

To find boundaries that ensure FWER control, an iterative search over the stopping boundaries is conducted based on the following inequality:

(5) $$\begin{array}{ll} \alpha \geq \sum_{w\in S}\left\{\sum_{k=1}^{K}\left[\int \text{⋯}\int_{A_{k}}p_{Z_{W}^{\left(1\right)},W}\left(z_{w}^{\left(1\right)},w;\Theta_{0}\right)\cdot \left(\prod_{m=k}^{1}p_{w,m|m-1}\left(z_{w}^{1:m}|z_{w}^{1:(m-1)};\Theta_{0}\right)\right)dz_{w}^{1:k}\text{⋯}dz_{w}^{\left(1\right)}\right]\right\}, & \end{array}$$

where the integration region *A*_*k*_

$$A_{k}=[C_{l_{1}},C_{u_{1},\alpha }]\times [C_{l_{2}},C_{u_{2},\alpha }]\times \text{⋯}\times [C_{u_{k},\alpha ,},\infty )\;\text{in}\;z_{w}^{\left(1\right)}\times z_{w}^{1:2}\text{⋯}\times z_{w}^{1:k},$$

where **Θ**_0_ denotes the globe null hypothesis. We define $z_{w}^{1:0}=z_{w}^{1:1}$ and therefore $p_{w,1|1}(z_{w}^{1:1}|z_{w}^{1:1};\Theta )=1$. Note that this yields only one inequality, whereas $C_{l_{1}},\text{⋯},C_{l_{k}}$ and $C_{u_{1},\alpha },\text{⋯},C_{u_{K},\alpha }$ are all unknown. To overcome this, we set them to follow a specific functional form, where $C_{l_{k}}=C_{u_{K},\alpha }$ for the *K* stage design. For example, when using the O'Brien Fleming (OBF)^13^, ^36^ type stopping boundaries, $C_{u_{k},\alpha }=C_{\text{OBF}}(K,\alpha )\sqrt{K/k}$ and $C_{l_{k}}$ is a certain function of *k*. In addition, the calculations in (5) assumes that the futility bounds are binding. For nonbinding bounds, one can simply set the lower bounds to −*∞*.

As before, (5) implies weak control of the FWER but also guarantees strong control following the arguments in the work of Magirr et al.^35^

Suppose an alternative hypothesis of the form *θ*_*w*_ = *δ* > 0 for exactly one element (say *w*) in S and $\theta_{w^{*}}=0\;\forall w^{*}\neq w\in S,$ is true. Then, under this alternative hypothesis, the aforementioned critical values, and specified power, the stage‐wise total sample size for the full population $n_{f}^{\left(k\right)}$ can be found to satisfy the following inequality:

(6) $$1-\beta \leq \sum_{k=1}^{K}\left[\int \text{⋯}\int_{A_{k}}p_{Z_{W}^{\left(1\right)},W}\left(z_{w}^{\left(1\right)},w;\Theta_{a}\right)\cdot \left(\prod_{m=k}^{1}p_{w,m|m-1}\left(z_{w}^{1:m}|z_{w}^{1:(m-1)};\Theta_{a}\right)\right)dz_{w}^{1:k}\text{⋯}dz_{w}^{\left(1\right)}\right],$$

where the configuration **Θ**_*a*_ has an nonzero positive effect *δ* on the *w*th element but the other *J* − 1 elements are zero. Detailed derivations of (5) and (6) are provided in Supplementary Materials S.1 and the design details of two‐stage designs with two subgroups (considering selection of *S*_1_ or *F*) in Supplementary Materials S.5.

#### 2.2.3 An illustrative example

The Dose Ranging Efficacy And safety with Mepolizumab in severe asthma (DREAM) trial^37^ investigates, among other endpoints, the effect of mepolizumab on exacerbations and forced expiratory volume in 1 second (FEV_1_). Subsequent secondary analyses of the trial data^38^, ^39^ find that the treatment effect of mepolizumab depends on the baseline levels of eosinophil and suggests that only patients with blood eosinophil levels of more than 150 cells per *μ*L receive benefit from the treatment.

Suppose that, on the basis of these exploratory findings, we wish to embark on a prospective evaluation of the claim that mepolizumab results in meaningful improvements only for patients with baseline levels of eosinophil of 150 or more cells per *μ*L in the blood. We will use a change in FEV_1_ from baseline to 90 days, modeled as normally distributed as the primary endpoints although the same arguments hold for other endpoints such as exacerbations. Additionally, we suppose that the prevalence of each group (below and above 150 cells per *μ*L blood) is 50%. Following the work of Santanello et al,^40^ we assume that the standard deviation is 0.72L and consider a reduction of FEV_1_ of 0.23L as the minimum clinically relevant treatment difference, and consequently seek to power our evaluations for this effect.

Three different evaluation strategies are considered, ie, (i) running two separate studies in each of the two subgroups, (ii) a single‐stage study with one subgroup versus the full population (see Section 2.2.1), and (iii) a two‐stage enrichment design where the best performing group is selected at the halfway point and early stopping using O'Brien and Flemming bounds^36^ are used (see Section 2.2.2). For each of the three designs, we consider a type I error per study of 2.5% and require a power of 80% to reject any false null hypothesis. Furthermore. we assume that 25 patients are recruited per month and that it takes two months to conduct the interim analysis for strategy 3.

A summary of the characteristics of the different strategies is given in Table 1. The strategy using two separate studies requires just over 600 patients to be recruited, whereas the single‐stage design with two groups does need almost 70 patients more. The reason for this is that no attempt has been made in the first approach to control the FWER. If we were to correct for multiplicity for the separate studies using a Bonferroni correction, the required sample size would increase to 748 patients. Using a two‐stage selection design allows us to reduce the required sample size even further to around 550 patients, a reduction of 10% and 30% as compared to the uncorrected and multiplicity corrected separate study strategy, respectively. Additionally, the two‐stage design does investigate more patients in the group that is truly benefitting from treatment, which is one of the reasons for the reduction in required sample size. Besides the reduction in sample size, running a single study rather than two separate ones does also yields organizational advantages. The main drawback of this approach is that the duration of the study is increased by almost nine months should the subgroup be selected (although a small reduction in the duration is expected if the full population is selected at interim).

**Table 1:** Comparison of different evaluation strategies. max family‐wise error rate (FWER) is the maximum family‐wise error of the strategy, N is the total sample size, % superior is the percentage of patient studied in the better performing subgroup, and duration is the time from recruiting the first patient until the primary endpoint is available for all patients

Strategy	max FWER	N	% superior	Duration (Months)
Separate studies	0.0494	616	50%	27.64
Single‐stage study	0.0250	684	50%	30.36
Two‐stage design	0.0250	552	75%	25.08 or 36.12

Note that, in addition to the advantages illustrated earlier, the FWER in the two‐stage enrichment design is controlled for the worst‐case situation in terms of selection and hence other selection rules can be used without error rate inflation.

#### 2.2.4 Alternative designs

We have illustrated how to obtain critical bounds and sample size for general enrichment designs earlier. Here, we discuss alternative designs considering different type‐I error and power configurations.

**Significance levels and stopping boundaries.**

An alternative to specifying the design and corresponding stage‐wise *α* levels via the boundaries is to specify marginal significance level *α*_*k*_ to each stage *k* (where $\sum_{k}\alpha_{k}=\alpha$) and use an error spending approach as used in classic group sequential designs.^13^ Such considerations affect the way we find stopping boundaries where the same boundaries are shared by all the populations considered. More specifically, based on the following inequality (7), it is required to search the critical value used in *A*_*k* − 1_ first under the upper limit of *α*_*k* − 1_ (where the subscript of the upper bounds is changed accordingly). Then, substitute those critical values for the associated bounds used in *A*_*k*_ under the upper limit of *α*_*k*_ for finding the remaining critical values and so on

(7) $$\sum_{i=1}^{k}\alpha_{i}\geq \sum_{w\in S}\left\{\left[\int \text{⋯}\int_{A_{k}}p_{Z_{W}^{\left(1\right)},W}\left(z_{w}^{\left(1\right)},w;\Theta_{0}\right)\cdot \left(\prod_{m=k}^{1}p_{w,m|m-1}\left(z_{w}^{1:m}|z_{w}^{1:(m-1)};\Theta_{0}\right)\right)dz_{w}^{1:k}\text{⋯}dz_{w}^{\left(1\right)}\right]\right\}.$$

Note that there are several ways to determine the lower stopping boundaries; for example, one could set symmetric values with respect to the upper critical values, or simply set 0.

One can further prespecify the marginal significance levels for |S|−1 specific populations at each stage. One example of taking this consideration can be found in the work of Spiessens and Debois,^9^ although they only consider single‐stage designs. Such design features may lead to different stopping boundaries for all the populations included in S.

Incidentally, for two‐stage designs, if early stopping is not considered at stage 1 (that is, the stage‐1 data is only used for population selection), then the first bound of integration in Equations (5) and (6), ie, *A*_*k*_, is ( −*∞*,*∞*), where *k* > 1. Meanwhile, the upper bound $C_{u_{1},\alpha_{1}}$ of *A*_1_ is defined as *∞* and therefore the integral $\int_{A_{1}}p_{Z_{W}^{\left(1\right)},W}(z_{w}^{\left(1\right)},w;\Theta_{0})dz_{w}^{\left(1\right)}$ is 0. Such designs are the same as the two‐stage adaptive seamless designs used in the work of Kimani et al.^31^

**Power.**

The power of the designs in Section 2.2 is defined as the probability to detect the treatment effect of the population of interest under *H*_*a*_. Alternatively, we can define power to detect any treatment effects wherever they are from a set of specific subpopulations. Such change leads the total sample size for *F* to be different because of its influence on Equation (6), which is the basis of searching $n_{f}^{\left(k\right)}$. Moreover, the equation becomes

(8) $$1-\beta \leq \sum_{w\in S^{*}}\left\{\sum_{k=1}^{K}\left[\int \text{⋯}\int_{A_{k}}p_{Z_{W}^{\left(1\right)},W}\left(z_{w}^{\left(1\right)},w;\Theta_{a}\right)\cdot \left(\prod_{m=k}^{1}p_{w,m|m-1}\left(z_{w}^{1:m}|z_{w}^{1:(m-1)};\Theta_{a}\right)\right)dz_{w}^{1:k}\text{⋯}dz_{w}^{\left(1\right)}\right]\right\},$$

where $S^{*}$ is the subset of S and contains the specified subpopulations of interest. Take an example that, if S={1,f} and $S^{*}=S$, Figure 1 shows the resulting total sample sizes $n_{f}^{\left(1\right)}$ in a single‐stage design, corresponding to different prevalence values of *S*_1_, under different definitions of power. The left panel is computed to have power 1 − *β* for selecting the subpopulation with the largest true effect and rejecting the corresponding null hypothesis, whereas the right panel considers any correct rejection. Under the left power definition, the required sample size is large when the prevalence of the subgroup with a positive treatment effect is small as the number of patients having said effect is (relatively) small. As the prevalence λ_1_ approaches 1, $n_{f}^{\left(1\right)}$ increases again as the effect of the subgroup dominates the effect in the full population and differentiating between the two populations becomes more difficult. In contrast, $n_{f}^{\left(1\right)}$ always decreases under the definition of power to detect *θ*_1_ > 0 or *θ*_*f*_ > 0. Since the effect sizes for *S*_1_ and *F* are close, it is difficult to select the correct subgroup and thus large sample sizes are needed. The reason that the behavior of $n_{f}^{\left(1\right)}$ is always decreasing for larger prevalences in the right panel is that there is no restriction on selecting a prespecified population and reporting the efficacy. The decreasing pattern can be similar to that using the closed testing procedure^41^ in a single‐stage design, where the total sample is available for investigating any subpopulation without considering selection. Note that all the patterns observed in Figure 1 emerge in a case of multistage designs as well (not shown in this paper).

## 3 ESTIMATION ASSESSMENT

In this section, we report a simulation study assessing the properties of MLEs. Note that, in the reported figures, different scales for the y‐axes are used to highlight patterns.

### 3.1 Simulation setup

In our evaluations, we specify the FWER, ie, *α*, as 0.025 and set the sample size for each scenario so that the power of the design is 1 − *β* = 80%. Our alternative hypothesis is that the treatment has an effect of 0.5 in *S*_1_, whereas the effect of the treatment is zero for all other subgroups. Therefore, the power aims to detect the nonzero effect in *S*_1_ (that is to reject *H*_01_) once the first subgroup is selected. The assumed common variance across subpopulations, ie, *σ* ^2^, is set to 1 and we use 1 000 000 simulation runs.

The designs we consider are a single‐stage design with two subpopulations (**Design 1**), a single‐stage design with three subpopulations (**Design 2**), and a two‐stage design with two subpopulations and three subpopulations (**Design 3** and **Design 4**, respectively), with an OBF upper stopping boundary and a fixed lower boundary of zero is used. We calculate the stopping boundaries and the total sample sizes for *F* based on (2) and (3) for single‐stage designs (and (5) and (6) for multistage designs). The sample sizes and critical values for each of the designs are given in Appendix A (Table A1‐A2). Based on these four designs, several scenarios are investigated, altering the design features such as prevalence.

Denote $\hat{\theta }$ as the naive MLE (that is not accounting for selection) for the parameter *θ*, then ${\hat{\theta }}_{f}$ and ${\hat{\theta }}_{s}$ represent the MLEs for the treatment effect of *F* and *S*_*s*_, respectively. The estimates can be calculated by $Z_{s}^{\left(k\right)}/I_{s}^{\left(k\right)}={\bar{Y}}_{s,T}^{\left(k\right)}-{\bar{Y}}_{s,C}^{\left(k\right)}$, where *s* ∈ {1,*f* } in scenarios for **Design 1** and *s* ∈ {1,1 + 2, *f* } in scenarios for **Design 2**. In multistage scenarios, the MLE estimates of ${\hat{\theta }}_{f}$ and ${\hat{\theta }}_{1}$ are calculated by $Z_{s}^{1:M}/I_{s}^{1:M}={\bar{Y}}_{s,T}^{1:M}-{\bar{Y}}_{s,C}^{1:M}$, where *s* ∈ {1,*f* } and *M* is the stage at which the study stops.

We define bias as bias( $\hat{\theta }$) = E( $\hat{\theta })-\theta$ and the mean squared error (MSE), MSE( $\hat{\theta }$) = E( ${\left(\hat{\theta }-\theta \right)}^{2}$) as performance measures for estimation assessment. As the sample size for the full population satisfies the aforementioned power requirement and varies across different prevalence, a standardized scale is used in the assessments (readers are referred to Supplementary Materials S.7 for details on the standardization). In our subsequent evaluations, we will consider three situations. Firstly, we consider the treatment effect estimator regardless of the population being selected or the hypothesis test being significant. Secondly, we consider only the estimators of the selected populations, which is expected to result in *selection bias*. The third situation considers *reporting bias* and, for this, we only consider only the treatment effect estimates of the selected population if the corresponding hypothesis test is significant. Implicitly, we are therefore considering that the outcome of a study is only reported (published) if it was significant. Note that, in the evaluations to follow, we refer to the selection bias as *Select* *S*_*w*_ and the reporting bias as *Select* *S*_*w*_ + *Reject* *H*_0*w*_, where *w* in S specifies the population chosen through a selection rule. In addition to the bias and MSE depending on which subgroup has been selected, we also report the family‐wise (FW) bias and MSE, ie, the bias and MSE averaged over all possible selections.

### 3.2 Scenarios for Design 1

Scenarios here cover different prevalence values of *S*_1_, λ_1_ varying from 0.05 to 0.95 in increments of 0.05. We illustrate the assessments for the scenarios under three configurations of different values of *θ*_1_ and *θ*_2_ in Figure 3‐4. Their horizontal axes are for the prevalence of *S*_1_, λ_1_, and the vertical axes of the row‐wise panels are for standardized bias, standardized $\sqrt{\text{MSE}}$, and simulation proportions (%).

**Figure 2:**
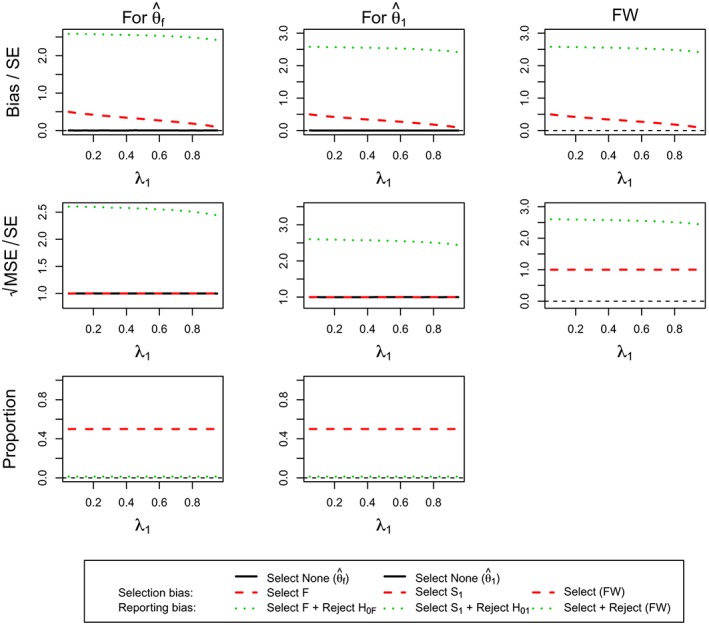
(For **Design 1**, θ_1_ = 0, and θ_2_ = 0) the standardized bias and standardized $\sqrt{\text{MSE}}$ of maximum likelihood estimators ${\hat{\theta }}_{f}$, ${\hat{\theta }}_{1}$ and the simulation proportions for different circumstances against the prevalence of subpopulation 1, ie, λ_1_. FW, family‐wise; MSE, mean squared error [Colour figure can be viewed at http://wileyonlinelibrary.com]

Figure 2 presents the estimation assessment of ${\hat{\theta }}_{f}$ and ${\hat{\theta }}_{1}$ under the assumption of *θ*_1_ = 0 and *θ*_2_ = 0. As expected, we do not see any bias when no selection is undertaken as well as constant standardized MSE, ie, a pattern that is repeated throughout all other simulations. Additionally, the selection probability is constant at 50% due to the equal effect in both subgroups. The selection bias is largest when the prevalence in the subgroup is smallest with a matching pattern for the standardized MSE. The reporting bias and MSE follow the same pattern although at a markedly increased level.

**Figure 3:**
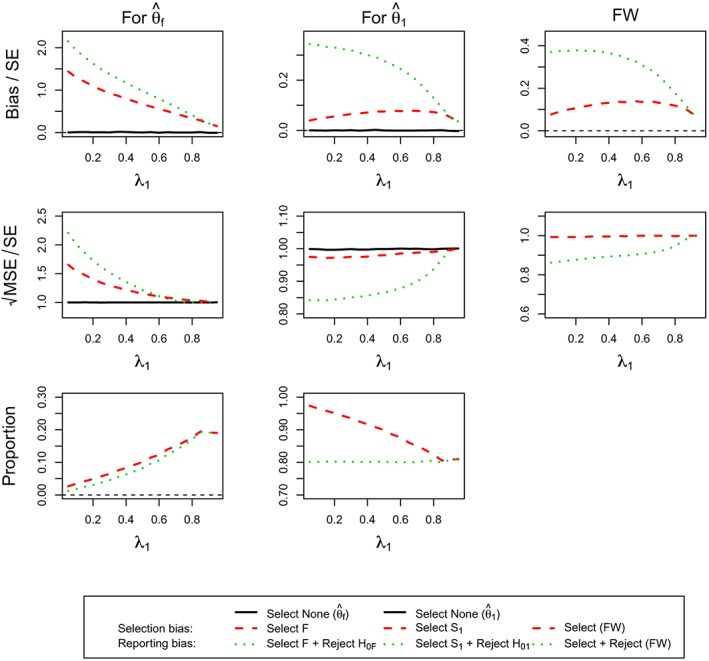
(For **Design 1**, θ_1_ = 0.5 and θ_2_ = 0) the standardized bias and standardized $\sqrt{\text{MSE}}$ of maximum likelihood estimators ${\hat{\theta }}_{f}$, ${\hat{\theta }}_{1}$ and the simulation proportions for different circumstances against the prevalence of subpopulation 1, λ_1_. FW, family‐wise; MSE, mean squared error [Colour figure can be viewed at http://wileyonlinelibrary.com]

Figure 3 considers the case when *θ*_1_ = 0.5 and *θ*_2_ = 0. Considering the selection probabilities first, we find that, as per design, there is an 80% chance to select population 1 correctly and reject the corresponding hypothesis. The selection probability of the full population increases as the prevalence increases as the effect in the full population gets larger as the subpopulation contributes more toward it. At the same time, the chance to also reject the hypothesis also increases. The selection and reporting bias in the full population estimate is largest when the prevalence in the subpopulation is smallest and then steadily decreases toward zero. The size of the bias is well over 0.5 standard errors for almost all prevalences and hence should be considered important although the incorrect selection in itself is not very common in this case. For the full population, the bias dominates the MSE and hence the MSE follows the same pattern.

Focusing the attention on subpopulation 1, we find that bias is present, although it is of much smaller magnitude (selection bias at most 0.1 and reporting bias at most 0.35 standard errors) than for the full population (up to over 2 standard errors). The selection bias is maximized at a prevalence of around 0.75, whereas it is largest for a small prevalence for the reporting bias.

**Figure 4:**
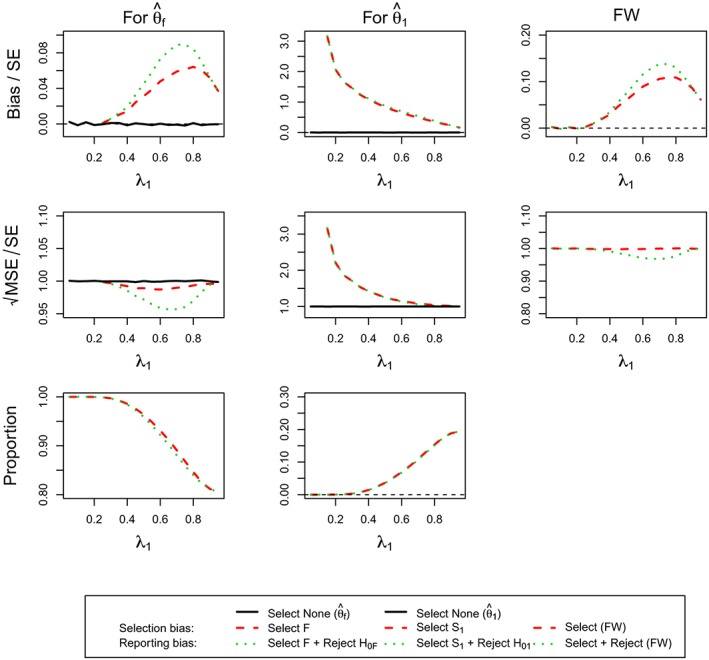
(For **Design 1**, θ_1_ = 0.5 and θ_2_ = 0.5) the standardized bias and standardized $\sqrt{\text{MSE}}$ of maximum likelihood estimators ${\hat{\theta }}_{f}$, ${\hat{\theta }}_{1}$ and the simulation proportions for different circumstances against the prevalence of subpopulation 1, ie, λ_1_. FW, family‐wise; MSE, mean squared error [Colour figure can be viewed at http://wileyonlinelibrary.com]

When both treatment groups have the same effect, *θ*_1_ = *θ*_2_ = 0.5 (Figure 4), we observe that, almost always, the full population is selected and only for large prevalences of the subpopulation (>50*%*) we obtain notable selection probability for the subpopulation (up to 20%). As a consequence of this, we obtain no estimate of the bias and MSE for the subpopulation for low prevalences. The bias in the estimate in this population is potentially very large (>3 standard errors) but drops quickly toward zero as the prevalence increases. In this setting, it is also notable that the selection bias is virtually identical to the reporting bias as very large observed effects are necessary to select the subpopulation in the first place.

The patterns for the full population are somewhat more distinct as no bias is observed for small prevalences because it is always the full population that is selected. The bias in this case is, however, very small even in the worst‐case situation (prevalence of around 0.75), where the reporting bias is less than 0.1 standard errors and the selection bias is even smaller.

### 3.3 Scenarios for Design 2

Scenarios for **Design 2** regard to select a population among *S*_1_, *S*_1 + 2_, and *F* under different configurations of *θ*_1_, *θ*_2_, and *θ*_3_. Our focus here is to assess the MLEs ${\hat{\theta }}_{1}$, ${\hat{\theta }}_{1}$, and ${\hat{\theta }}_{f}$ under *θ*_1_ = 0.5,*θ*_2_ = 0,*θ*_3_ = 0 under the population selection rule given by

(9) $$\left\{\begin{array}{cc} \text{select}\;S_{1},\; & \text{if}\;Z_{1}^{\left(k\right)}>\max\left(Z_{f}^{\left(k\right)},Z_{1+2}^{\left(k\right)}\right)\\ \text{select}\;S_{1+2}, & \text{if}\;Z_{1}^{\left(k\right)}≯\max\left(Z_{f}^{\left(k\right)},Z_{1+2}^{\left(k\right)}\right)\;,\;\text{and}\;Z_{1+2}^{\left(k\right)}>Z_{f}^{\left(k\right)}\\ \text{select}\;F,\; & \text{if}\;Z_{1}^{\left(k\right)}≯\max\left(Z_{f}^{\left(k\right)},Z_{1+2}^{\left(k\right)}\right)\;,\;\text{and}\;Z_{1+2}^{\left(k\right)}<Z_{f}^{\left(k\right)}. \end{array}\right.$$

This rule is one variant of the maximum statistic rule and sequentially decides which population to be selected. The results for other configurations of *θ*_1_, *θ*_2_, and *θ*_3_ are provided in Tables S.1‐S.3 in Supplementary Materials S.8. Note that, for all the scenarios, simulations are run under the same stopping boundaries and sample sizes ( $n_{f}^{\left(1\right)}=576$) found based on **Design 2** with the maximum statistics selection rule, *θ*_1_ = 0.5,*θ*_2_ = 0,*θ*_3_ = 0, and equal subgroup prevalence.

The results in Table 2 shows that, in this case, the correct population is selected most of the time (>80*%*) due to the design constraint to obtain 80% power. The selection bias when selecting the correct population is small at <0.1 standard errors and even the reporting bias is only modest at 0.27 standard errors. The selection and reporting bias when selecting the incorrect population are notably larger in this instance resulting in biases up to 1.3 standard errors. The bias is largest for the full population as the true underlying effect in this group is at 0.167 smallest among all populations and hence a rather unusual sample is required for its MLE to be the largest.

**Table 2:** (For **Design 2**, θ_1_ = 0.5, θ_2_ = 0, and θ_3_ = 0) Standardized bias and standardized $\sqrt{\text{MSE}}$ of the maximum likelihood estimators where the prevalence rates of three subgroups are 1/3. In addition, Proportion (Prop.) stands for how often the corresponding circumstance occurs

	Bias/SE	$\sqrt{\text{MSE}}/\text{SE}$	Prop.(%)
${\hat{\theta }}_{f}$ (Select none)	‐0.00186	0.99849	
${\hat{\theta }}_{f}$ (Select F)	0.96546	1.32104	3.74
${\hat{\theta }}_{f}$ (Select F + reject *H*_0*F*_)	1.31217	1.47472	2.91
${\hat{\theta }}_{1}$ (Select none)	‐0.00151	1.00004	
${\hat{\theta }}_{1}$ (Select *S*_1_)	0.09094	0.97526	88.58
${\hat{\theta }}_{1}$ (Select *S*_1_ + reject *H*_01_)	0.27068	0.87036	80.20
${\hat{\theta }}_{1+2}$ (Select none)	‐0.00118	0.99884	
${\hat{\theta }}_{1+2}$ (Select *S*_1+2_)	0.76128	1.19617	7.68
${\hat{\theta }}_{1+2}$ (Select *S*_1+2_ + reject *H*_0,1+2_)	1.02579	1.26021	6.47
Family‐wise select	0.17516	1.00518	
Family‐wise select + reject	0.35902	0.91814

Abbreviations: MSE, mean squared error.

### 3.4 Scenarios for Design 3

The investigation presented here concerns Design 3, a two‐stage design and we focus on *θ*_1_ = 0.5 and *θ*_2_ = 0 here, whereas the results for other configurations are given in Figures S.1‐S.6 of Supplementary Materials S.8.

**Figure 5:**
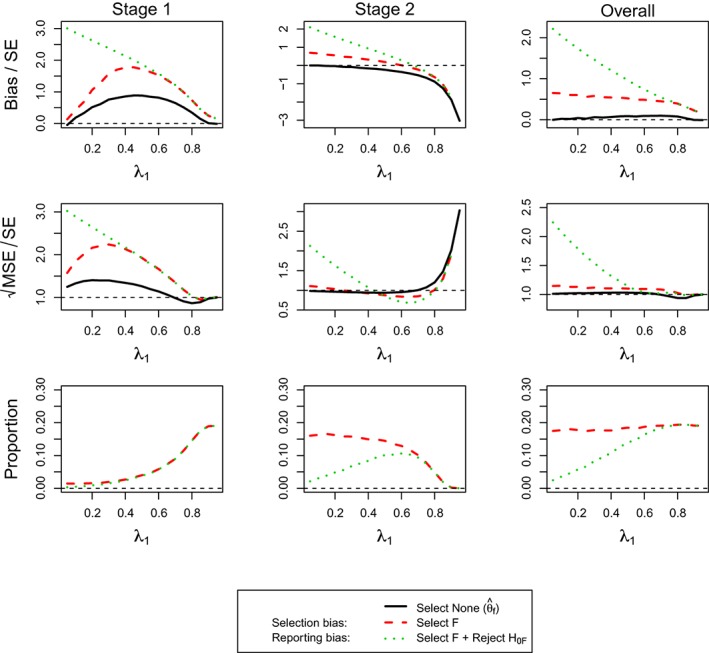
(For **Design 3**, θ_1_ = 0.5 and θ_2_ = 0) standardized bias and mean squared error (MSE) of ${\hat{\theta }}_{f}$ and simulation proportions for different circumstances at stopping stage 1, 2, and overall, against the prevalence of subpopulation 1, ie, λ_1_ [Colour figure can be viewed at http://wileyonlinelibrary.com]

Figure 5 shows the results of the estimator for the full population. The top row corresponds to standardized bias, middle row to standardized $\sqrt{\text{MSE}}$, and the bottom row to the probability of selecting the full population. The first column is associated with the estimators that stop at Stage 1, the second considers only trials that reach Stage 2, whereas the final column corresponds to the estimator irrespective of when the trial was stopped. In addition to the selection bias and the reporting bias, we also consider the estimator irrespective of the reason for stopping (green triangle) in the figure.

The reporting bias is potentially very large (up to three standard errors for Stage 1 only and up to two standard errors for Stage 2) and is the largest when the prevalence of the subgroup is small and subsequently decreases. When only considering studies that select the full population and stop at Stage 1, it approaches zero, whereas the bias does in fact become negative for trials that stop at the second stage. The overall estimator is, however, always positively biased, showing a very similar pattern as the Stage 1 cases only. The selection bias overall and, for Stage 2, only follows the same pattern as the reporting bias, whereas it does show an inverted U‐shape for Stage 1 only, which is maximized at a prevalence of around 0.5. The bias in the estimator that only considers stopping at Stage 1 for any reason follows the same pattern as the selection bias, although the bias is smaller. It is noteworthy that, although substantial bias is exhibited under some situation, the probability of reaching these (eg, selecting the full population and stopping at Stage 1) are very rare. The standardized $\sqrt{\text{MSE}}$ appears like that in standardized bias except for the second stage. In those exceptional cases, the MSE (for selection, reporting, and regardless of selection) decreases at a different rate before inflating substantially at a prevalence of 0.8.

**Figure 6:**
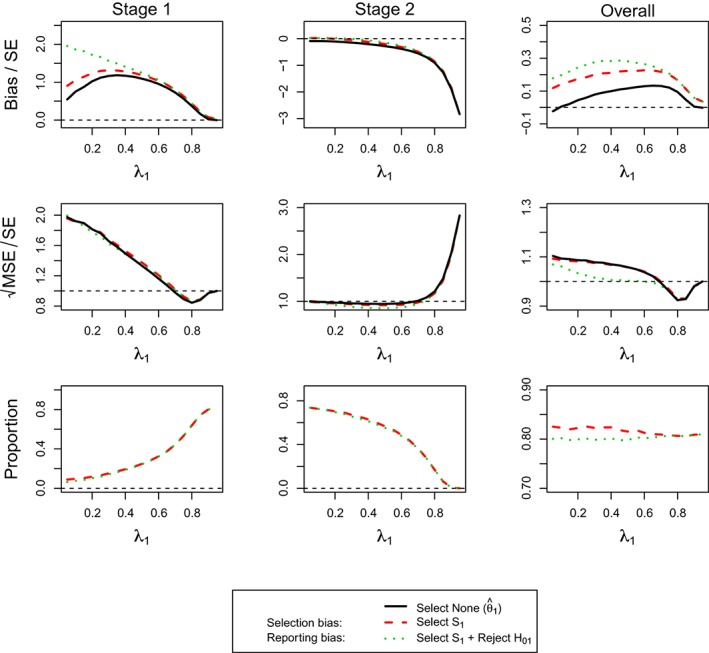
(For **Design 3**, θ_1_ = 0.5 and θ_2_ = 0) standardized bias and mean squared error (MSE) of ${\hat{\theta }}_{1}$ and simulation proportions for different circumstances at stopping stage 1, 2, and overall, against the prevalence of subpopulation 1, ie, λ_1_ [Colour figure can be viewed at http://wileyonlinelibrary.com]

Considering the findings for the estimator of the first subpopulation, ie, ${\hat{\theta }}_{1}$ (Figure 6), the results exhibit similar patterns in many circumstances in Figure 5. When stopping the trial at the first stage, the estimator is largely biased for prevalences up to 0.6. The reporting bias subsequently decreases from two standard errors, whereas the selection bias is more moderate at around 1 SE. All the MSE (regardless of any circumstances) decreases to 0.9 SE from 2 and is close one for larger prevalances larger 0.7. As most of the time, the subpopulation is selected correctly, the selection bias and the bias considering all studies that stopped at Stage 1 are very similar and the MSE, meanwhile, is near one standard error. The estimators considering only trials that stop at Stage 2 are almost unbiased for small and moderate prevalence but can exhibit a large negative bias when the prevalence is large. The MSE is close to 1 SE for most of prevalences but becomes very large beyond a prevalence of 0.7. The overall estimator is, however, positively biased (for both selection and reporting) for all prevalences and shows an inverted U‐shape with a maximum bias of about 0.3 SEs for a prevalence of 0.6. Its MSE conditional on selection or no‐selection appears different from that considering reporting before a prevalence of 0.7. The estimator thereafter performs similarly in MSE with a small U‐shape under 1 SE.

**Figure 7:**
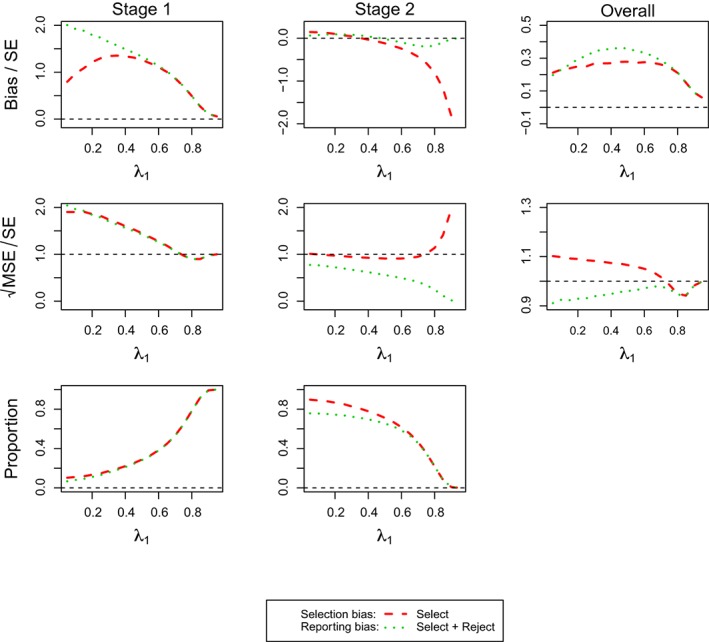
(For **Design 3**, θ_1_ = 0.5 and θ_2_ = 0) FW bias and mean squared error and simulation proportions for different circumstances at stopping stage 1, 2, and overall, against the prevalence of subpopulation 1, ie, λ_1_ [Colour figure can be viewed at http://wileyonlinelibrary.com]

The FW bias and MSE for this design with *θ*_1_ = 0.5 and *θ*_2_ = 0 are given in Figure 7.

### 3.5 Scenarios for Design 4

Scenarios for **Design 4** is the two‐stage counterpart of **Design 2** for selecting a population among *S*_1_, *S*_1+2_, and *F* under different configurations of *θ*_1_, *θ*_2_, and *θ*_3_. The investigation here focus on assessing the maximum likelihood estimators ${\hat{\theta }}_{1}$, ${\hat{\theta }}_{1+2}$, and ${\hat{\theta }}_{f}$ under *θ*_1_ = 0.5,*θ*_2_ = 0,*θ*_3_ = 0 under the population selection rule given in Equation 9. The results for other configurations of *θ*_1_, *θ*_2_, and *θ*_3_ are provided in Tables S.4‐S.6 in Supplementary Materials S.8. All the simulations are run under the same stopping boundaries and sample sizes ( $n_{f}^{\left(1\right)}=335$) found based on **Design 4** with the maximum statistics selection rule, the configuration of treatment effects (*θ*_1_ = 0.5,*θ*_2_ = 0,*θ*_3_ = 0) and subgroup prevalences being 1/3.

Table 3 shows the results of the estimators for the first subgroup, the combined subgroup, and the full population. The standardized bias, standardized $\sqrt{\text{MSE}}$, and simulation proportions are presented in the trials that stop at Stage 1, reach Stage 2, and are irrespective of which stopping stage.

**Table 3:** For **Design 4**, θ_1_ = 0.5, θ_2_ = 0, and θ_3_ = 0) standardized bias and standardized $\sqrt{\text{MSE}}$ of the MLEs where the prevalence rates of three subgroups are 1/3. In addition, Proportion (Prop.) stands for how often the corresponding circumstance occurs

	Stop at Stage 1			Stop at Stage 2			Overall		
	Bias/SE	$\sqrt{\text{MSE}}/\text{SE}$	Prop.(%)	Bias/SE	$\sqrt{\text{MSE}}/\text{SE}$	Prop.(%)	Bias/SE	$\sqrt{\text{MSE}}/\text{SE}$	Prop.(%)
${\hat{\theta }}_{f}$ (Select none)	0.67715	1.13140		‐0.26340	0.96585		0.05614	1.02209	
${\hat{\theta }}_{f}$ (Select F )	2.02110	2.14318	1.54	0.36337	0.92461	5.98	0.70283	1.17414	7.52
${\hat{\theta }}_{f}$ (Select F + reject *H*_0*F*_)	2.10568	2.14995	1.51	0.85268	1.01727	3.89	1.20283	1.33379	5.39
${\hat{\theta }}_{1}$ (Select none)	1.03255	1.22555		‐0.29968	0.97840		0.15293	1.06237	
${\hat{\theta }}_{1}$ (Select *S*_1_ )	1.12932	1.27694	29.13	‐0.20496	0.94416	51.17	0.27906	1.06487	80.29
${\hat{\theta }}_{1}$ (Select *S*_1_ + reject *H*_01_)	1.14828	1.26420	28.99	‐0.20175	0.93853	51.11	0.28684	1.05639	80.11
${\hat{\theta }}_{1+2}$ (Select none)	0.81892	1.16958		‐0.26809	0.96219		0.10120	1.03265	
${\hat{\theta }}_{1+2}$ (Select *S*_1+2_)	1.78983	1.88884	3.31	0.17732	0.89687	8.88	0.61488	1.16604	12.18
${\hat{\theta }}_{1+2}$ (Select *S*_1+2_ + reject *H*_0,1+2_)	1.82834	1.88619	3.27	0.41744	0.81323	7.57	0.84338	1.13715	10.85
Family‐wise select	1.23403	1.37576	33.97	‐0.10207	0.93603	66.03	0.35185	1.08542	
Family‐wise select + reject	1.25693	1.36402	33.77	‐0.06135	0.92826	62.57	0.40076	1.08101	

Abbreviations: MSE, mean squared error.

Considering the trials irrespective of stopping, we observed the correct population is selected in the 80% of simulations due to the design requirement of 80% power. The bias is found positive for all the overall estimators and varies widely (smallest at 0.05 and maximum up to 1.2 standard errors). The selection and reporting bias when selecting the correct population are the smallest (less than 0.3 standard errors), but larger when selecting the incorrect population (particularly for the full population). All the standardized MSE are larger than one standard error but only up to a moderate size of around 1.3. While selecting the correct population or rejecting the null hypothesis, the estimator for the first subgroup has a smaller standardized MSE (around 1.06 standard errors) than its counterparts.

The results at different stages show a contrary picture. More trials stop at Stage 2 than at Stage 1 and each stage has a higher proportion of selecting the correct population (around 30% and 50% at Stage 1 and Stage 2, respectively). The bias is large at Stage 1. The selection and reporting bias are smaller when selecting *S*_1_ (around 1.1 standard errors) than those when selecting *S*_1+2_ or *F* (around 1.8 and 2, respectively). A moderate bias is observed at Stage 2 (up to 0.85 standard errors). In particular, the selection and reporting bias are found negative in the estimator for the first subgroup. The standardized MSE of all the estimators at Stage 1 are much larger than one SE but those at Stage 2 show the opposite pattern, being less than 1 (between 0.8 and 1).

## 4 DISCUSSIONS AND CONCLUDING REMARKS

In this paper, we have discussed general design considerations for clinical trials with subpopulation selection and illustrate how such studies can be designed. The design framework described can be viewed as an extension of group‐sequential methods^42^ and therefore requires the same types of assumptions and specifically we do assume an independent increment structure of the data. In our evaluations, we have assumed that the primary endpoint is available immediately or at least before the next patient is recruited to the trial. While the general results in the paper will remain to hold if the endpoint is available only after some time, patients may still be recruited from a subpopulation that is subsequently not selected. Different approaches to deal with delayed responses have been proposed (eg, the work of Hampson and Jennison^43^) in the context of group‐sequential trials have been proposed. As a general rule, however, it is clear that the efficiency of selection is reduced if the time to observe the endpoint is long in comparison with the recruitment speed. Other assumptions made within this framework are common to most adaptive designs. Most notably, we are assuming that there are differences in the population before and after interim analysis and, in particular, that no time trends are present.

In this work, we only consider designs with normally distributed endpoints, although they can easily be extended to other types of endpoints via the efficient scores framework.^42^, ^44^ Note, however, that particular care is required when using time to event endpoints (see the work of Magirr et al}^45^ for a more detailed challenges of adaptive trials with time to event endpoints). Moreover, we assume that the subgroup prevalence is known although clearly specifying this parameter correctly in the design will be crucial for the designs operating characteristics. A consequence of the assumed known prevalence is that we only present the estimation assessment of the MLE, where subgroup sample sizes are fixed according to the respective prevalence in designs. Further simulations (not shown), however, suggest that random sample sizes of populations only alter the findings marginally.

Selection based on the maximum test statistics is the main focus throughout the paper and an R package implementing this design is currently under development. While this selection rule is simple and intuitive, it may not be optimal in certain circumstances. It makes sense to adopt the rule when some subgroup treatment effects have been identified as being positive and difference between test statistics across subgroups are reasonably large. However, when the test statistic for *S*_*s*_ and *F* are close but the former is larger, applying this rule leads to ethical issues that selecting only part of the population rather than the whole population although they could benefit from the treatment. Therefore, other options for selection rules should be considered for similar situations and investigation.

One alternative, which is also considered for designs with treatment selection (eg, see the work of Bretz et al^46^), can be to introduce a threshold in the selection rule. This allows all the subgroups whose effect sizes are similar to the best one (their absolute difference is within a threshold) to be united so that the pooled population can continue to the next stage. Meanwhile, it also permits to select a population whose effect size is above a threshold plus the effect size from the others.

Another option that has been used in the context of treatment selection (eg, see the works of Magnusson and Turnbull^16^ and Magirr and Jaki^35^ is simply to select a population whose efficacy exceeds a certain value at stage 1. This selection rule was used in the work of Magnusson and Turnbull^16^ and integrates population selection and hypothesis testing at the first stage. Their designs considering a prior ordering on underlying effect sizes of all individual subgroups somehow connect to ours where the target subpopulations for selection has a nested structure. It is noted that the mathematical expression of $p_{Z_{W}^{\left(1\right)},W}(\cdot ,\cdot )$ in (1) will be different if the aforementioned selection rules are used. We provide the required modifications to the design framework in the supplementary materials for illustrative purposes.

In term of estimation, we have assessed the bias of the MLE under various scenarios. We find that, almost always, bias is positive leading to an overenthusiastic estimate of the true treatment effect. While for some settings, the size of the bias can be viewed as negligible, it can become large under other situations. The challenge clearly being that one will usually not know if one is in one of these extreme situations. Another observation we make is that, although bias is introduced by selecting the population, the bias gets markedly increased (often more than doubled) when only significant results are reported highlighting the effect of reporting bias, which may be even more problematic than the bias introduced by selection.

Our results suggest that the MSE of the overall MLEs performs quite well (around 1 standard error) in many circumstances and scenarios. We find whether selecting the correct population or not impacts the size of MSE for the corresponding estimator. The extent can be more substantial when further reporting significant results. The same finding is even observed in the extreme scenario, where no correct population is defined because the underlying effect of each subgroup is assumed none.

Future work will consider estimators that are unbiased (or have smaller bias) while maintaining comparable MSE. The conditional bias‐adjusted estimator following the ideas in the work of Stallard and Todd^28^ appears as the most promising. One extension to the case of multiple‐stage designs given the process continues to the final stage can be naturally achieved. However, whether the derived estimators have less MSE should be verified in further investigations.

## Supporting information

SIM_7925‐Supp‐0001‐SIMPaper_SM_V2.pdf

## APPENDIX A

### DESIGN SPECIFICATIONS

#### A.1 Design 1

**Table A1:** Design specifications for different values of λ for Design 1. c is the critical value and N is the total sample size

λ	0.05	0.1	0.15	0.2	0.25	0.3	0.35	0.4	0.45	0.5
*c*	2.232	2.228	2.223	2.217	2.212	2.206	2.200	2.193	2.186	2.178
*N*	3070	1546	1040	788	638	539	469	418	380	351
λ	0.55	0.6	0.65	0.7	0.75	0.8	0.85	0.9	0.95	
*c*	2.170	2.160	2.150	2.139	2.126	2.111	2.094	2.072	2.042	
*N*	329	313	303	298	302	318	363	493	943	

#### A.2 Design 2

**Table A2:** Design specifications for different values of λ for Design 2 and an interim analysis after half the patients have been observed. c_1_ is the upper stopping boundary at stage 1, c_2_ the final stage critial value, and N the total sample size. The lower bound at stage 1 is fixed at zero for all λ

λ	0.05	0.1	0.15	0.2	0.25	0.3	0.35	0.4	0.45	0.5
*c*_1_	3.018	3.031	3.037	3.039	3.039	3.037	3.034	3.029	3.023	3.016
*c*_2_	2.134	2.143	2.147	2.149	2.149	2.148	2.145	2.142	2.138	2.133
*N*	719	401	298	251	224	207	196	188	183	181
λ	0.55	0.6	0.65	0.7	0.75	0.8	0.85	0.9	0.95	
*c*_1_	3.008	2.999	2.989	2.977	2.964	2.948	2.930	2.907	2.875	
*c*_2_	2.127	2.121	2.114	2.105	2.096	2.085	2.072	2.055	2.033	
*N*	181	184	192	205	229	269	342	491	943	

#### A.3 Design 3

For this single‐stage design with three subgroups, the prevalance of each subgroup is equal to one third, resulting in a critical value of *c* = 2.289 and a total sample size of *N* = 575.

#### A.4 Design 4

The two stage design with three subgroups uses equal prevalance of each subgroup and an interim analysis after half the patients have been observed. The critical value at the first stage is *c*_1_ = 3.119, whereas the final critical value is *c*_2_ = 2.205. A fixed futility bound of zero is used and the total sample size is *N* = 335.
