# Supplementary material for: Design and estimation in clinical trials with subpopulation selection
Source: Stat Med. 2018 Aug 7;37(29):4335–52. doi: 10.1002/sim.7925 (PMC6282861; doi:10.1002/sim.7925)
Supplement: Supplementary file 1 — SIM_7925‐Supp‐0001‐SIMPaper_SM_V2.pdf [file SIM-37-4335-s001.pdf]

Research Article

Statistics in Medicine

Received XXXX

(www.interscience.wiley.com) DOI: 10.1002/sim.0000

SUPPLEMENTARY MATERIAL: Design and estimation in clinical trials with subpopulation selection

Yi-Da Chiu[a], Franz Koenig[b], Martin Posch[b] and Thomas Jaki[a*†]

S.1. The derivations of Stopping Boundaries and Sample Size

Consider all the possible situations that lead to rejecting any individual null hypothesis, then the inequality (2) of the main paper for searching critical values can be derived based on

$$\begin{aligned}\alpha \geq Pr\Big[\bigcup_{w\in\mathcal{S}} (Z_W^{(1)} > C_\alpha, W = w) \,|\, H_0\Big] &= \sum_{w\in\mathcal{S}} Pr\Big[(Z_W^{(1)} > C_\alpha, W = w) \,|\, H_0\Big] \\ = \sum_{w\in\mathcal{S}} \int_{C_\alpha}^{\infty} p_{Z_W^{(1)},W}(z_w^{(1)}, w; \boldsymbol{\Theta}_0) dz_w^{(1)} & \qquad \text{(S.1)}\end{aligned}$$

and similarly equation (3) of the main paper which can be used to find the total sample size is based on

$$\begin{aligned}1-\beta \geq Pr\Big[(Z_W^{(1)} > C_\alpha, W = w) \,|\, H_a\Big] &= Pr\Big[(Z_W^{(1)} > C_\alpha, W = w) \,|\, H_a\Big] \\ = \int_{C_\alpha}^{\infty} p_{Z_W^{(1)},W}(z_w^{(1)}, w; \boldsymbol{\Theta}_a) dz_w^{(1)}. & \qquad \text{(S.2)}\end{aligned}$$

[a] Medical and Pharmaceutical Statistics Research Unit, Department of Mathematics and Statistics, Lancaster University, LA1 4YF, U.K.
[b] Center for Medical Statistics, Informatics, and Intelligent Systems, Medical University of Vienna, Spitalgasse 23, 1090 Vienna, Austria.
[*] Correspondence to: Thomas Jaki, Medical and Pharmaceutical Statistics Research Unit, Department of Mathematics and Statistics, Lancaster University, LA1 4YF, U.K.
[†] E-mail: t.jaki@lancaster.ac.uk

Copyright © 2017 John Wiley & Sons, Ltd.

For a multi-stage setting we begin equally by considering all situations that lead to rejecting any individual null hypothesis at any stages and find equation (5) of the main paper as

$$\begin{aligned}
\alpha \geq\; & Pr\Big[\bigcup_{w\in\mathcal{S}}(Z_W^{(1)} > C_{u_1,\alpha}, W=w)\,|\,H_0\Big] + Pr\Big[\bigcup_{w\in\mathcal{S}}(C_{l_1} < Z_W^{(1)} < C_{u_1,\alpha}, Z_W^{1:2} > C_{u_2,\alpha}, W=w)\,|\,H_0\Big]\\
& + \ldots + Pr\Big[\bigcup_{w\in\mathcal{S}}(C_{l_1} < Z_W^{(1)} < C_{u_1,\alpha}, \ldots, C_{l_{K-1}} < Z_W^{1:(K-1)} < C_{u_{K-1},\alpha}, Z_W^{1:K} > C_{u_K,\alpha}, W=w)\,|\,H_0\Big]\\
=& \sum_{w\in\mathcal{S}}\Bigg\{Pr\Big[(Z_W^{(1)} > C_{u_1,\alpha}, W=w)\,|\,H_0\Big] + Pr\Big[(C_{l_1} < Z_W^{(1)} < C_{u_1,\alpha}, Z_W^{1:2} > C_{u_2,\alpha}, W=w)\,|\,H_0\Big]\\
& + \ldots + Pr\Big[(C_{l_1} < Z_W^{(1)} < C_{u_1,\alpha}, \ldots, C_{l_{K-1}} < Z_W^{1:(K-1)} < C_{u_{K-1},\alpha}, Z_W^{1:K} > C_{u_K,\alpha}, W=w)\,|\,H_0\Big]\Bigg\}\\
=& \sum_{w\in\mathcal{S}}\Bigg[\int_{C_{u_1,\alpha}}^{\infty} p_{Z_W^{(1)},W}(z_w^{(1)}, w; \mathbf{\Theta}_0)dz_w^{(1)} + \int_{C_{l_1}}^{C_{u_1,\alpha}}\int_{C_{u_2,\alpha}}^{\infty} p_{Z_W^{(1)},W}(z_w^{(1)}, w; \mathbf{\Theta}_0)\cdot p_{w,2|1}(z_w^{1:2}|z_w^{(1)}; \mathbf{\Theta}_0)dz_w^{1:2}dz_w^{(1)}\\
& + \ldots + \int_{C_{l_1}}^{C_{u_1,\alpha}}\cdots\int_{C_{l_{K-1}}}^{C_{u_{K-1},\alpha}}\int_{C_{u_K,\alpha}}^{\infty} p_{Z_W^{(1)},W}(z_w^{(1)}, w; \mathbf{\Theta}_0)\cdot p_{w,2|1}(z_w^{1:2}|z_w^{(1)})\cdot\cdots\\
& \cdots p_{w,K|K-1}(z_w^{1:K}|z_w^{1:(K-1)})dz_w^{1:K}dz_w^{1:(K-1)}\ldots dz_w^{(1)}\Bigg]\\
=& \sum_{w\in\mathcal{S}}\Bigg\{\sum_{k=1}^{K}\Big[\int\cdots\int_{A_k} p_{Z_W^{(1)},W}(z_w^{(1)}, w; \mathbf{\Theta}_0)\cdot\Big(\prod_{m=k}^{1} p_{w,m|m-1}(z_w^{1:m}|z_w^{1:(m-1)}; \mathbf{\Theta}_0)\Big)dz_w^{1:k}\ldots dz_w^{(1)}\Big]\Bigg\}.
\end{aligned} \tag{S.3}$$

Similarly the inequality (6) of the main paper is found as

$$\begin{aligned}
1-\beta \leq\; & Pr\Big[(Z_W^{(1)} > C_{u_1,\alpha}, W=w)\,|\,H_a\Big] + Pr\Big[(C_{l_1} < Z_W^{(1)} < C_{u_1,\alpha}, Z_W^{1:2} > C_{u_2,\alpha}, W=w)\,|\,H_a\Big]\\
& + \ldots + Pr\Big[(C_{l_1} < Z_W^{(1)} < C_{u_1,\alpha}, \ldots, C_{l_{K-1}} < Z_W^{1:(K-1)} < C_{u_{K-1},\alpha}, Z_W^{1:K} > C_{u_K,\alpha}, W=w)\,|\,H_a\Big]\\
=& Pr\Big[(Z_W^{(1)} > C_{u_1,\alpha}, W=w)\,|\,H_a\Big] + Pr\Big[(C_{l_1} < Z_W^{(1)} < C_{u_1,\alpha}, Z_W^{1:2} > C_{u_2,\alpha}, W=w)\,|\,H_a\Big]\\
& + \ldots + Pr\Big[(C_{l_1} < Z_W^{(1)} < C_{u_1,\alpha}, \ldots, C_{l_{K-1}} < Z_W^{1:(K-1)} < C_{u_{K-1},\alpha}, Z_W^{1:K} > C_{u_K,\alpha}, W=w)\,|\,H_a\Big]\\
=& \int_{C_{u_1,\alpha}}^{\infty} p_{Z_W^{(1)},W}(z_w^{(1)}, w; \mathbf{\Theta}_a)dz_w^{(1)} + \int_{C_{l_1}}^{C_{u_1,\alpha}}\int_{C_{u_2,\alpha}}^{\infty} p_{Z_W^{(1)},W}(z_w^{(1)}, w; \mathbf{\Theta}_a)\cdot p_{w,2|1}(z_w^{1:2}|z_w^{(1)}; \mathbf{\Theta}_a)dz_w^{1:2}dz_w^{(1)}\\
& + \ldots + \int_{C_{l_1}}^{C_{u_1,\alpha}}\cdots\int_{C_{l_{K-1}}}^{C_{u_{K-1},\alpha}}\int_{C_{u_K,\alpha}}^{\infty} p_{Z_W^{(1)},W}(z_w^{(1)}, w; \mathbf{\Theta}_a)\cdot p_{w,2|1}(z_w^{1:2}|z_w^{(1)})\cdot\cdots\\
& \cdots p_{w,K|K-1}(z_w^{1:K}|z_w^{1:(K-1)})dz_w^{1:K}dz_w^{1:(K-1)}\ldots dz_w^{(1)}\\
=& \sum_{k=1}^{K}\Big[\int\cdots\int_{A_k} p_{Z_W^{(1)},W}(z_w^{(1)}, w; \mathbf{\Theta}_a)\cdot\Big(\prod_{m=k}^{1} p_{w,m|m-1}(z_w^{1:m}|z_w^{1:(m-1)}; \mathbf{\Theta}_a)\Big)dz_w^{1:k}\ldots dz_w^{(1)}\Big].
\end{aligned} \tag{S.4}$$

S.2. Design 1 - single-stage designs with two subgroups

Given the index set for population selection $\mathcal{S} = \{1, f\}$, the joint distribution of two test statistics $Z_1^{(1)}$ and $Z_f^{(1)}$ is

$$\begin{pmatrix} Z_1^{(1)} \\ Z_f^{(1)} \end{pmatrix} = \mathcal{N}\left(\begin{pmatrix} \theta_1 I_1^{(1)} \\ \theta_f I_f^{(1)} \end{pmatrix}, \begin{pmatrix} 1 & \sqrt{\lambda_1} \\ \sqrt{\lambda_1} & 1 \end{pmatrix}\right).$$

 Copyright © 2017 John Wiley & Sons, Ltd.

Let the selected test statistic and the selected population index be $Z_W^{(1)}$ and W. Both are random variables and particularly $W = 1$ or f refers to whether subpopulation 1 or the full population is chosen. The joint density of $Z_W^{(1)}$ and W, $p_{Z_W^{(1)},W}(z_w^{(1)}, w)$, is equal to $p_{Z_w^{(1)}}(z_w^{(1)}) \cdot p(W = w|Z_w^{(1)} = z_w^{(1)})$. If $Z_w^{(1)} = z_w^{(1)}$, then $W = w$ if $Z_u^{(1)} < z_w^{(1)}$ for all $u \neq w$. To express the probability for this event it is required to find the distribution of $Z_u^{(1)}|Z_w^{(1)} = z_w^{(1)}$. As $Z_u^{(1)}$ and $Z_w^{(1)}$ are correlated, we need to exploit a property of conditional densities of the multivariate normal distribution (refer to Section 0.3 in [1]. Applying this fact it follows that

$$Z_f^{(1)}|Z_1^{(1)} = z_1^{(1)} \sim \mathcal{N}\left(\theta_f I_f^{(1)} + \sqrt{\lambda_1}(z_1^{(1)} - \theta_1 I_1^{(1)}), 1 - \lambda_1\right),$$

and

$$Z_1^{(1)}|Z_f^{(1)} = z_f^{(1)} \sim \mathcal{N}\left(\theta_1 I_1^{(1)} + \sqrt{\lambda_1}(z_f^{(1)} - \theta_f I_f^{(1)}), 1 - \lambda_1\right).$$

Then the densities of the joint distribution of $Z_W^{(1)}$ and W are

$$p_{Z_W^{(1)},W}(z_w^{(1)}, w; \boldsymbol{\Theta}) = \phi(z_w^{(1)} - \theta_w I_w^{(1)})\Phi\Big(\frac{z_w^{(1)} - \theta_u I_u^{(1)} - \sqrt{\lambda_1}(z_w^{(1)} - \theta_w I_w^{(1)})}{\sqrt{1-\lambda_1}}\Big), \quad w \neq u \in \{1, f\}.$$

As a result, equation (2) for critical value C_α and equation (3) in the main paper for the total sample size of F are respectively as follows:

$$\begin{aligned} \alpha &\geq \int_{C_\alpha}^{\infty} p_{Z_W^{(1)},W}(z_1^{(1)}, 1; \boldsymbol{\Theta}_0)dz_1^{(1)} + \int_{C_\alpha}^{\infty} p_{Z_W^{(1)},W}(z_f^{(1)}, f; \boldsymbol{\Theta}_0)dz_f^{(1)}, \\ 1 - \beta &\leq \int_{C_\alpha}^{\infty} p_{Z_W^{(1)},W}(z_1^{(1)}, 1; \boldsymbol{\Theta}_a)dz_1^{(1)}. \end{aligned}$$

S.3. Design 2 - single-stage designs with three subgroups

Define the index set for population selection as $\mathcal{S} = \{1, 1+2, f\}$, the joint distribution of three test statistics $Z_1^{(1)}$, $Z_{1+2}^{(1)}$ and $Z_f^{(1)}$ is then

$$\begin{pmatrix} Z_1^{(1)} \\ Z_{1+2}^{(1)} \\ Z_f^{(1)} \end{pmatrix} = \mathcal{N}\left(\begin{pmatrix} \theta_1 I_1^{(1)} \\ \theta_{1+2} I_{1+2}^{(1)} \\ \theta_f I_f^{(1)} \end{pmatrix}, \begin{pmatrix} 1 & \sqrt{\frac{\lambda_1}{\lambda_1+\lambda_2}} & \sqrt{\lambda_1} \\ \sqrt{\frac{\lambda_1}{\lambda_1+\lambda_2}} & 1 & \sqrt{\lambda_1+\lambda_2} \\ \sqrt{\lambda_1} & \sqrt{\lambda_1+\lambda_2} & 1 \end{pmatrix}\right),$$

where the entries of the above covariance matrix are obtained by

$$cov(Z_1^{(1)}, Z_{1+2}^{(1)}) = \frac{I_1^{(1)}}{I_{1+2}^{(1)}}, \quad cov(Z_1^{(1)}, Z_f^{(1)}) = \frac{I_1^{(1)}}{I_f^{(1)}}, \quad cov(Z_{1+2}^{(1)}, Z_f^{(1)}) = \frac{I_{1+2}^{(1)}}{I_f^{(1)}}.$$

To derive the densities of the joint distribution of $Z_W^{(1)}$ and W, the conditional densities of $Z_u^{(1)}, Z_v^{(1)}|Z_w^{(1)} = z_w^{(1)}$ for all $u, v \neq w$ need to be found. Using the gaussian identity about the conditional densities of the multivariate normal distribution, they are

$$(Z_{1+2}^{(1)}, Z_f^{(1)})^\mathsf{T}|Z_1^{(1)} = z_1^{(1)} \sim \mathcal{N}(\mu_{1+2,f}, \Sigma_{1+2,f}), \quad \text{(S.5)}$$

$$(Z_1^{(1)}, Z_f^{(1)})^\mathsf{T}|Z_{1+2}^{(1)} = z_{1+2}^{(1)} \sim \mathcal{N}(\mu_{1,f}, \Sigma_{1,f}), \quad \text{(S.6)}$$

$$(Z_1^{(1)}, Z_{1\cup2}^{(1)})^\mathsf{T}|Z_f^{(1)} = z_f^{(1)} \sim \mathcal{N}(\mu_{1+2}, \Sigma_{1,1+2}), \quad \text{(S.7)}$$

where

$$\mu_{1+2,f} = \begin{pmatrix} \theta_{1+2} I_{1+2}^{(1)} + \sqrt{\frac{\lambda_1}{\lambda_1+\lambda_2}}(z_1^{(1)} - \theta_1 I_1^{(1)}) \\ \theta_f I_f^{(1)} + \sqrt{\lambda_1}(z_1^{(1)} - \theta_1 I_1^{(1)}) \end{pmatrix},$$

$$\mu_{1,f} = \begin{pmatrix} \theta_1 I_1^{(1)} + \sqrt{\frac{\lambda_1}{\lambda_1+\lambda_2}}(z_{1+2}^{(1)} - \theta_{1+2} I_{1+2}^{(1)}) \\ \theta_f I_f^{(1)} + \sqrt{\lambda_1+\lambda_2}(z_{1+2}^{(1)} - \theta_{1+2} I_{1+2}^{(1)}) \end{pmatrix},$$

$$\mu_{1,1+2} = \begin{pmatrix} \theta_1 I_1^{(1)} + \sqrt{\lambda_1}(z_f^{(1)} - \theta_f I_f^{(1)}) \\ \theta_{1+2} I_{1+2}^{(1)} + \sqrt{\lambda_1+\lambda_2}(z_f^{(1)} - \theta_f I_f^{(1)}) \end{pmatrix},$$

and

$$\Sigma_{1+2,f} = \begin{pmatrix} 1 & \sqrt{\lambda_1+\lambda_2} \\ \sqrt{\lambda_1+\lambda_2} & 1 \end{pmatrix} - \begin{pmatrix} \sqrt{\frac{\lambda_1}{\lambda_1+\lambda_2}} \\ \sqrt{\lambda_1} \end{pmatrix} \begin{pmatrix} \sqrt{\frac{\lambda_1}{\lambda_1+\lambda_2}} & \sqrt{\lambda_1} \end{pmatrix},$$

$$\Sigma_{1,f} = \begin{pmatrix} 1 & \sqrt{\lambda_1} \\ \sqrt{\lambda_1} & 1 \end{pmatrix} - \begin{pmatrix} \sqrt{\frac{\lambda_1}{\lambda_1+\lambda_2}} \\ \sqrt{\lambda_1+\lambda_2} \end{pmatrix} \begin{pmatrix} \sqrt{\frac{\lambda_1}{\lambda_1+\lambda_2}} & \sqrt{\lambda_1+\lambda_2} \end{pmatrix},$$

$$\Sigma_{1,1+2} = \begin{pmatrix} 1 & \sqrt{\frac{\lambda_1}{\lambda_1+\lambda_2}} \\ \sqrt{\frac{\lambda_1}{\lambda_1+\lambda_2}} & 1 \end{pmatrix} - \begin{pmatrix} \sqrt{\lambda_1} \\ \sqrt{\lambda_1+\lambda_2} \end{pmatrix} \begin{pmatrix} \sqrt{\lambda_1} & \sqrt{\lambda_1+\lambda_2} \end{pmatrix},$$

As a result, the joint densities of $Z_W^{(1)}$ and W with different elements of W are

$$p_{Z_W^{(1)},W}(z_w^{(1)}, w; \boldsymbol{\Theta}) = \phi(z_w^{(1)} - \theta_w I_w^{(1)})\Psi_{u,v}\Big(z_w^{(1)}, z_w^{(1)}; \boldsymbol{\Theta}\Big), \qquad u, v \neq w \in \{1, 1+2, f\},$$

where $\Psi_{u,v}(\cdot,\cdot;\boldsymbol{\Theta})$ is the conditional cumulative distribution function of the bivariate normal distribution corresponding to (A.5) or (A.6) or (A.7). Consequently, equation (2) of the main paper for critical value C_α is as follows:

$$\alpha \geq \int_{C_\alpha}^{\infty} p_{Z_W^{(1)},W}(z_1^{(1)}, 1; \boldsymbol{\Theta}_0) dz_1^{(1)} + \int_{C_\alpha}^{\infty} p_{Z_W^{(1)},W}(z_{1+2}^{(1)}, 1+2; \boldsymbol{\Theta}_0) dz_{1+2}^{(1)} + \int_{C_\alpha}^{\infty} p_{Z_W^{(1)},W}(z_f^{(1)}, f; \boldsymbol{\Theta}_0) dz_f^{(1)}.$$

And the equation which searching sample sizes depends on is the same as that in Section A.2.

S.4. The derivations of conditional densities (multi-stage designs with multiple subgroups)

The conditional densities in equation (4) of the main paper are derived from the definition of the test statistic and the distributional properties. Given S_w being chosn ($w \in \mathcal{S}$), the test statistics based on the accumulated data at the end of stage k, $Z_w^{1:k}$, can also be written as

$$Z_w^{1:k} = \sum_{i=1}^{k} \sqrt{\frac{I_w^{(i)}}{I_w^{1:k}}} Z_w^{(i)} = \sum_{i=1}^{k} \sqrt{\frac{n_w^{(i)}}{n_w^{1:k}}} Z_w^{(i)} = \sqrt{\frac{I_w^{1:(k-1)}}{I_w^{1:k}}} Z_w^{1:(k-1)} + \sqrt{\frac{I_w^{(k)}}{I_w^{1:k}}} Z_w^{(k)} = \sqrt{\frac{n_w^{1:(k-1)}}{n_w^{1:k}}} Z_w^{1:(k-1)} + \sqrt{\frac{n_w^{(k)}}{n_w^{1:k}}} Z_w^{(k)},$$

Then,

$$Z_w^{1:k} - \sqrt{\frac{n_w^{1:(1-k)}}{n_w^{1:k}}} Z_w^{1:(1-k)} \quad \sim \quad \mathcal{N}\Big(\sqrt{\frac{n_w^{(k)}}{n_w^{1:k}}} I_w^{(k)} \theta_w, \frac{n_w^{(k)}}{n_w^{1:k}}\Big),$$

 Copyright © 2017 John Wiley & Sons, Ltd.
Prepared using simauth.cls

so that the conditional distribution of $Z_w^{1:k}$ given $Z_w^{1:(k-1)} = z_w^{1:(k-1)}$ follows

$$Z_w^{1:k}|Z_w^{1:(k-1)} = z_w^{1:(k-1)} \quad \sim \quad \mathcal{N}\Big(\sqrt{\frac{n_w^{1:(k-1)}}{n_w^{1:k}}} z_w^{1:(k-1)} + \sqrt{\frac{n_w^{(k)}}{n_w^{1:k}}} I_w^{(k)} \theta_w, \frac{n_w^{(k)}}{n_w^{1:k}}\Big).$$

The conditional densities with different population selection are

$$p_{w,k|k-1}(Z_w^{1:k}|Z_w^{1:(k-1)} = z_w^{1:(k-1)}; \mathbf{\Theta}) = \frac{1}{\sqrt{\frac{n_w^{(k)}}{n_w^{1:k}}}} \phi\Bigg(\frac{Z_w^{1:k} - \sqrt{\frac{n_w^{1:(1-k)}}{n_w^{1:k}}} z_w^{1:(k-1)} - \sqrt{\frac{n_w^{(k)}}{n_w^{1:k}}} I_w^{(k)} \theta_w}{\sqrt{\frac{n_w^{(k)}}{n_w^{1:k}}}}\Bigg), \tag{S.8}$$

In general, $\frac{n_w^{1:(k-1)}}{n_w^{1:k}} = \frac{\lambda_w + (k-2)}{\lambda_w + (k-1)}$ and $\frac{n_w^{(k)}}{n_w^{1:k}} = \frac{1}{\lambda_w + (k-1)}$, where λ_w is the prevalence of S_w in the full population F, where $w \in \mathcal{S}$.

S.5. Design 3 - two-stage designs with two subgroups

Given the index set for population selection $\mathcal{S} = \{1, f\}$, Equation (5) of the main paper for critical values is:

$$\alpha \geq \int_{C_{u_1,\alpha}}^{\infty} p_{Z_W^{(1)},W}(z_1^{(1)}, 1; \mathbf{\Theta}_0) dz_1^{(1)} + \int_0^{C_{u_1,\alpha}} \int_{C_{u_2,\alpha}}^{\infty} p_{Z_W^{(1)},W}(z_1^{(1)}, 1; \mathbf{\Theta}_0) \cdot p_{w,2|1}(z_1^{1:2}|z_1^{(1)}; \mathbf{\Theta}_0) dz_1^{1:2} dz_1^{(1)} +$$
$$\int_{C_{u_1,\alpha}}^{\infty} p_{Z_W^{(1)},W}(z_f^{(1)}, f; \mathbf{\Theta}_0) dz_f^{(1)} + \int_0^{C_{u_1,\alpha}} \int_{C_{u_2,\alpha}}^{\infty} p_{Z_W^{(1)},W}(z_f^{(1)}, f; \mathbf{\Theta}_0) \cdot p_{w,2|1}(z_f^{1:2}|z_f^{(1)}; \mathbf{\Theta}_0) dz_f^{1:2} dz_f^{(1)},$$

where $C_{u_1,\alpha}$ and $C_{u_2,\alpha}$ are upper critical values at stage 1 and 2. Moreover, $C_{u_1,\alpha} = C_{OBF}(2,\alpha)\sqrt{2}$ and $C_{u_2,\alpha} = C_{OBF}(2,\alpha)$. In addition, C_{l_1} and C_{l_2} are zero at stage 1 and 2.

The stagewise total sample size $n_f^{(k)}$ can be found with critical values and specified power. More clearly, under the alternative hypothesis H_a,

$$1 - \beta \leq \int_{C_{u_1,\alpha}}^{\infty} p_{Z_W^{(1)},W}(z_1^{(1)}, 1; \mathbf{\Theta}_a) dz_1^{(1)} + \int_0^{C_{u_1,\alpha}} \int_{C_{u_2,\alpha}}^{\infty} p_{Z_W^{(1)},W}(z_1^{(1)}, 1; \mathbf{\Theta}_a) \cdot p_{w,2|1}(z_1^{1:2}|z_1^{(1)}; \mathbf{\Theta}_a) dz_1^{1:2} dz_1^{(1)}.$$

S.6. Joint distribution on the basis of a threshold rule

Let $\mathcal{S}$ be the index set of the population for selection. In particular, if subgroup 1, or subgroup 1 and 2 or the full population are of interest, $\mathcal{S} = \{1, 1+2, f\}$. If $\mathcal{S}$ includes the index of nested subgroups, it can alternatively be rewritten as the index set for disjoint subgroups. Then the threshold selection rule (TSR) for designs with subgroup selection is

Select S_w if

$$Z_w^{(1)} > C. \tag{S.9}$$

and combined subgroups (e.g. S_w and $S_{w'}$) are selected if

$$Z_w^{(1)} > C, \; Z_{w'}^{(1)} > C, \qquad w \neq w^{'} \in \mathcal{S}. \tag{S.10}$$

Copyright © 2017 John Wiley & Sons, Ltd.

For **TSR**, the joint density $p(\mathbf{Z}_W^{(1)}, W; \mathbf{\Theta})$ has the form

$$p_{Z_W^{(1)},W}(z_w^{(1)}, w; \mathbf{\Theta}) = h_w(z_w^{(1)}; \mathbf{\Theta}) Pr(W = w; \mathbf{\Theta}), \tag{S.11}$$

where $h_w(\cdot; \mathbf{\Theta})$ is the pdf of a truncated normal distribution of $Z_w^{(1)}$ over the interval (C, ∞). $Pr(W = w; \mathbf{\Theta})$ is the probability of selecting the subgroup S_w. Note that if S_w is a combined subgroup, the pdf of the truncated normal distribution is the convolution of the truncated normal pdfs of the components. For example if S_{1+2} has been selected (that is, w = 1+2), then

$$h_{1+2}(z_{1+2}^{(1)}; \mathbf{\Theta}) = \int_{-\infty}^{\infty} h_2(z_{1+2}^{(1)} - z_1^{(1)}; \mathbf{\Theta}) h_1(z_1^{(1)}; \mathbf{\Theta}) dz_1^{(1)}.$$

S.7. Standardization Procedures of Estimation Assessment Measures in Section 3.2-3.5

Bias and mean squared error (MSE) are converted to a standardized scale because some cases consider varying λ_1 and have different sample sizes for the full population across the prevalence. In Section 3.2 (scenarios for *Design* 1), for $\hat{\theta}_1$, the standardization is undertaken through multiplying bias($\hat{\theta}_1$) and $\sqrt{\text{MSE}(\hat{\theta}_1)}$ by $I_1^{(1)} = 1/\big(\sigma\sqrt{1/n_{1,T}^{(1)} + 1/n_{1,C}^{(1)}}\big)$; and for $\hat{\theta}_f$, multiplying bias($\hat{\theta}_f$) and $\sqrt{\text{MSE}(\hat{\theta}_f)}$ by $I_f^{(1)} = 1/\big(\sigma\sqrt{1/n_{f,T}^{(1)} + 1/n_{f,C}^{(1)}}\big)$.

In Section 3.3 (scenarios for *Design* 2), the standardization procedures of bias and MSE for $\hat{\theta}_1$ and $\hat{\theta}_f$ are the same as those in Design 1. For $\hat{\theta}_{1+2}$, multiplying bias($\hat{\theta}_{1+2}$) and $\sqrt{\text{MSE}(\hat{\theta}_{1+2})}$ by $I_{1+2}^{(1)} = 1/\big(\sigma\sqrt{1/n_{1+2,T}^{(1)} + 1/n_{1+2,C}^{(1)}}\big)$.

Due to the same reason as that in Section 3.2, we transform bias into a standardized scale in scenarios for *Design* 3. At stage 1 the standardization procedures of bias and $\sqrt{\text{MSE}}$ for $\hat{\theta}_1$ and $\hat{\theta}_f$ are the same as those in *Design* 1. At stage 2, the standardization for $\hat{\theta}_f$ is performed by multiplying the corresponding information level (based on the accumulated data to stage 2). But for $\hat{\theta}_1$, due to the enrichment design feature the standardization needs to consider different information levels according to the outcomes of population selection. More specifically, if F is selected at stage 1, then the standardization can be fulfilled by multiplying the information level $I_f^{1:2} = 1/\big(\sigma\sqrt{1/n_{f,T}^{1:2} + 1/n_{f,C}^{1:2}}\big) = 1/\big(\sigma\sqrt{1/(n_{f,T}^{(1)} + n_{f,T}^{(2)}) + 1/(n_{f,C}^{(1)} + n_{f,C}^{(2)})}\big)$. If S_1 is selected at stage 1, then the standardization multiplicative factor is $I_1^{1:2} = 1/\big(\sigma\sqrt{1/n_{1,T}^{1:2} + 1/n_{1,C}^{1:2}}\big) = 1/\big(\sigma\sqrt{1/(\lambda_1 n_{f,T}^{(1)} + n_{f,T}^{(2)}) + 1/(\lambda_1 n_{f,C}^{(1)} + n_{f,C}^{(2)})}\big)$.

Regardless of population selection or hypothesis testing, the standardized bias and $\sqrt{\text{MSE}}$ at stage 2 can be obtained by multiplying a weighted average of the accumulated information levels (considering whether F or S_1 is selected at stage 1). The weights are the proportions of selecting F or S_1 over all the simulations stopping at stage 2.

The standardization procedure of bias and $\sqrt{\text{MSE}}$ in Design 4 mixes those in Design 2 and Design 3. It incorporates the information levels (including the accumulated ones) of the estimator for S_{1+2}.

S.8. Other Scenarios

The following figures provide an overview of estimation assessments and simulation proportions for other scenarios under **Design 2**, **Design 3** and **Design 4**.

 Copyright © 2017 John Wiley & Sons, Ltd.

	Bias/SE	$\sqrt{\text{MSE}}$/SE	Prop.(%)
$\hat{\theta}_f$ (Select None)	0.00070	1.00111	
$\hat{\theta}_f$ (Select F)	0.61007	1.13018	6.73
$\hat{\theta}_f$ (Select F + Reject H_{0F})	0.62751	1.12185	6.69
$\hat{\theta}_1$ (Select None)	-0.00048	1.00082	
$\hat{\theta}_1$ (Select S_1)	0.89487	1.28668	2.88
$\hat{\theta}_1$ (Select S_1 + Reject H_{01})	0.92464	1.28359	2.84
$\hat{\theta}_{1+2}$ (Select None)	0.00045	1.00075	
$\hat{\theta}_{1+2}$ (Select S_{1+2})	0.06905	0.98314	90.39
$\hat{\theta}_{1+2}$ (Select S_{1+2} + Reject $H_{0,1+2}$)	0.07748	0.97220	90.13
Family-wise Select	0.12927	1.00179	
Family-wise Select + Reject	0.13859	0.99114	

Table S.1. (For **Design 2**, $\theta_1 = 0.5$, $\theta_2 = 0.5$ and $\theta_3 = 0$) Standardized bias and standardized $\sqrt{\text{MSE}}$ of the MLEs where the prevalence rates of three subgroups are 1/3. In addition, Proportion (Prop.) stands for how often the corresponding circumstance occurs.

	Bias/SE	$\sqrt{\text{MSE}}$/SE	Prop.(%)
$\hat{\theta}_f$ (Select None)	0.00087	1.00005	
$\hat{\theta}_f$ (Select F)	0.02751	0.99165	96.37
$\hat{\theta}_f$ (Select F + Reject H_{0F})	0.02776	0.99118	96.36
$\hat{\theta}_1$ (Select None)	0.00007	0.99936	
$\hat{\theta}_1$ (Select S_1)	1.45101	1.71585	0.22
$\hat{\theta}_1$ (Select S_1 + Reject H_{01})	1.45374	1.71607	0.21
$\hat{\theta}_{1+2}$ (Select None)	0.00055	0.99999	
$\hat{\theta}_{1+2}$ (Select S_{1+2})	0.67728	1.17471	3.41
$\hat{\theta}_{1+2}$ (Select S_{1+2} + Reject $H_{0,1+2}$)	0.67819	1.17400	3.40
Family-wise Select	0.05272	0.99945	
Family-wise Select + Reject	0.05299	0.99897	

Table S.2. (For **Design 2**, $\theta_1 = 0.5$, $\theta_2 = 0.5$ and $\theta_3 = 0.5$) Standardized bias and standardized $\sqrt{\text{MSE}}$ of the MLEs where the prevalence rates of three subgroups are 1/3. In addition, Proportion (Prop.) stands for how often the corresponding circumstance occurs.

	Bias/SE	$\sqrt{\text{MSE}}$/SE	Prop.(%)
$\hat{\theta}_f$ (Select None)	-0.00035	1.00077	
$\hat{\theta}_f$ (Select F)	0.44103	1.03131	34.53
$\hat{\theta}_f$ (Select F + Reject H_{0F})	2.64275	2.66308	0.86
$\hat{\theta}_1$ (Select None)	-0.00003	1.00001	
$\hat{\theta}_1$ (Select S_1)	0.43384	1.02366	38.71
$\hat{\theta}_1$ (Select S_1 + Reject H_{01})	2.64200	2.66239	0.91
$\hat{\theta}_{1+2}$ (Select None)	-0.00097	1.00021	
$\hat{\theta}_{1+2}$ (Select S_{1+2})	0.50868	1.06019	26.76
$\hat{\theta}_{1+2}$ (Select S_{1+2} + Reject $H_{0,1+2}$)	2.64538	2.66543	0.76
Family-wise Select	0.45636	1.03609	
Family-wise Select + Reject	2.64328	2.66354	

Table S.3. (For **Design 2**, $\theta_1 = 0$, $\theta_2 = 0$ and $\theta_3 = 0$) Standardized bias and standardized $\sqrt{\text{MSE}}$ of the MLEs where the prevalence rates of three subgroups are 1/3. In addition, Proportion (Prop.) stands for how often the corresponding circumstance occurs.

Copyright © 2017 John Wiley & Sons, Ltd.

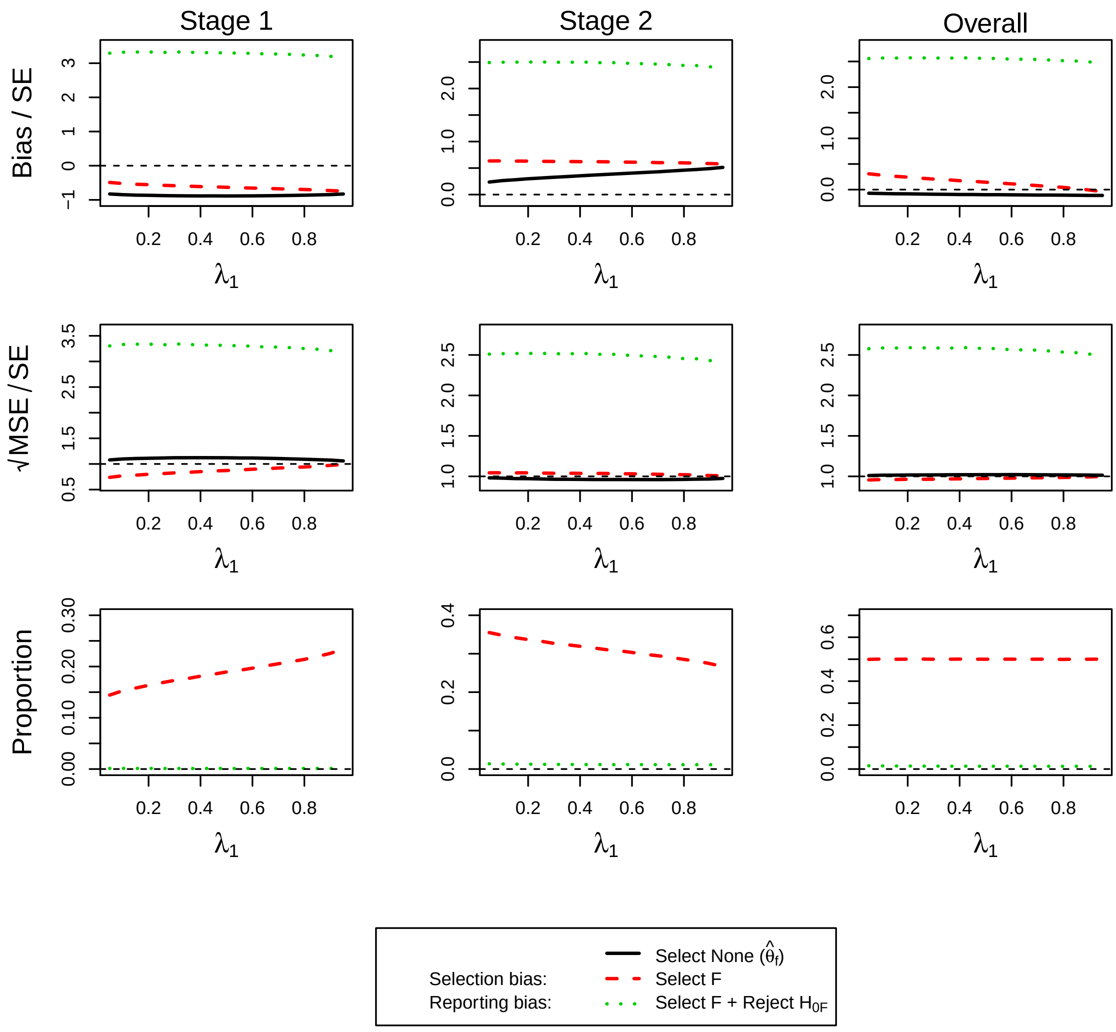

Figure S.1. (For **Design 3**, $\theta_1 = 0$ and $\theta_2 = 0$) standardized bias and MSE of $\hat{\theta}_f$ and simulation proportions for different circumstances at stopping stage 1, 2 and overall, against the prevalence of subpopulation 1, λ_1.

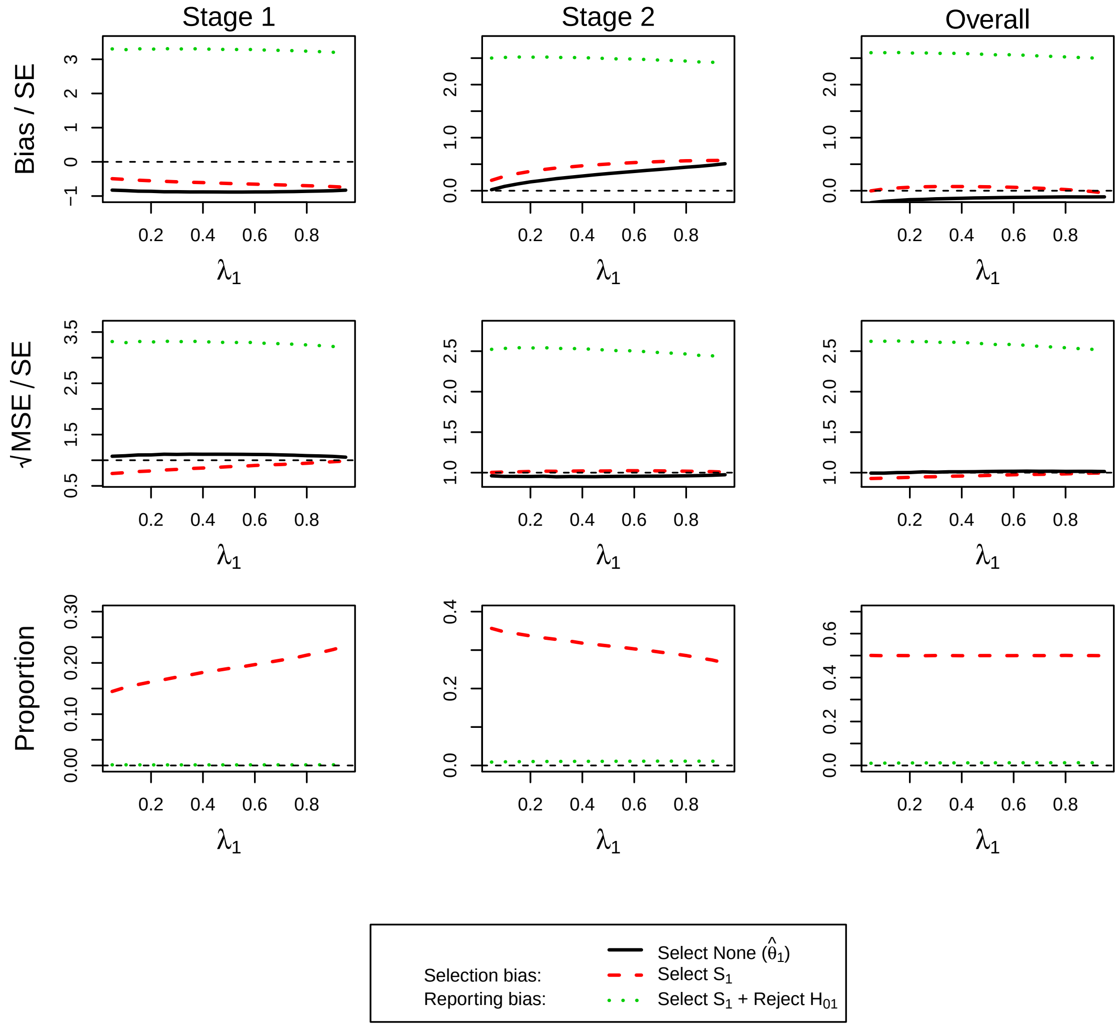

Figure S.2. (For **Design 3**, $\theta_1 = 0$ and $\theta_2 = 0$) standardized bias and MSE of $\hat{\theta}_1$ and simulation proportions for different circumstances at stopping stage 1, 2 and overall, against the prevalence of subpopulation 1, λ_1.

Copyright © 2017 John Wiley & Sons, Ltd.

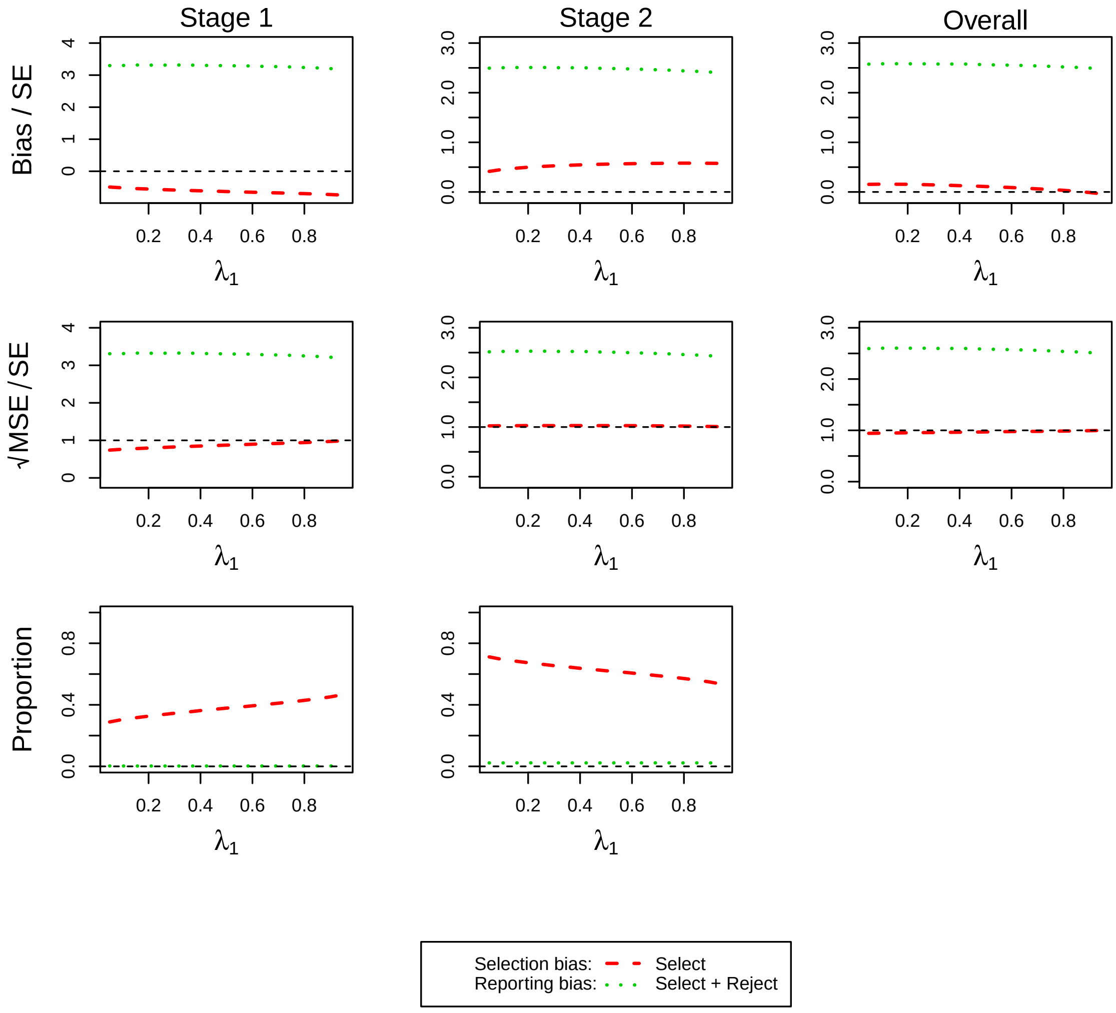

Figure S.3. (For **Design 3**, $\theta_1 = 0$ and $\theta_2 = 0$) family-wise bias and MSE and simulation proportions for different circumstances at stopping stage 1, 2 and overall, against the prevalence of subpopulation 1, λ_1.

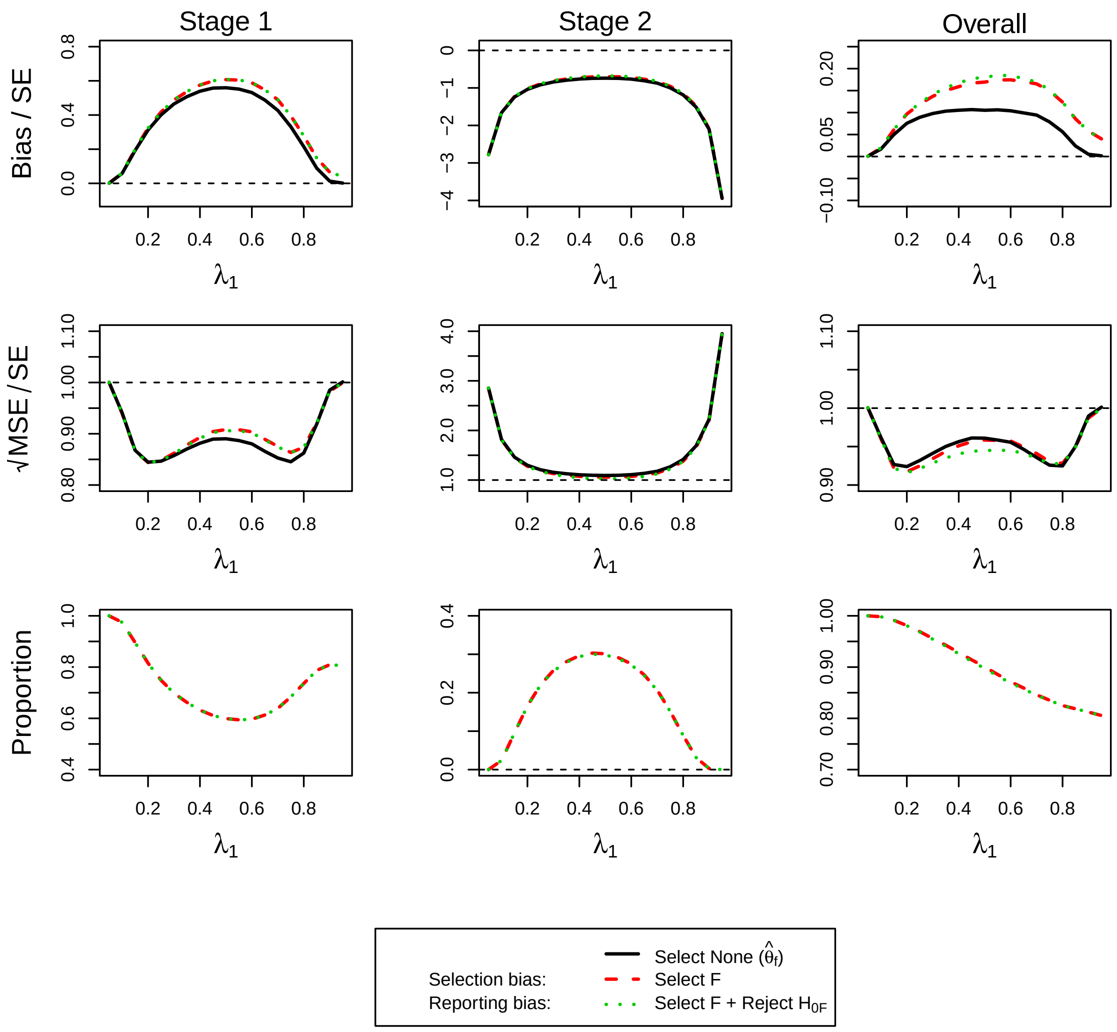

Figure S.4. (For **Design 3**, $\theta_1 = 0.5$ and $\theta_2 = 0.5$) standardized bias and MSE of $\hat{\theta}_f$ and simulation proportions for different circumstances at stopping stage 1, 2 and overall, against the prevalence of subpopulation 1, λ_1.

Copyright © 2017 John Wiley & Sons, Ltd.

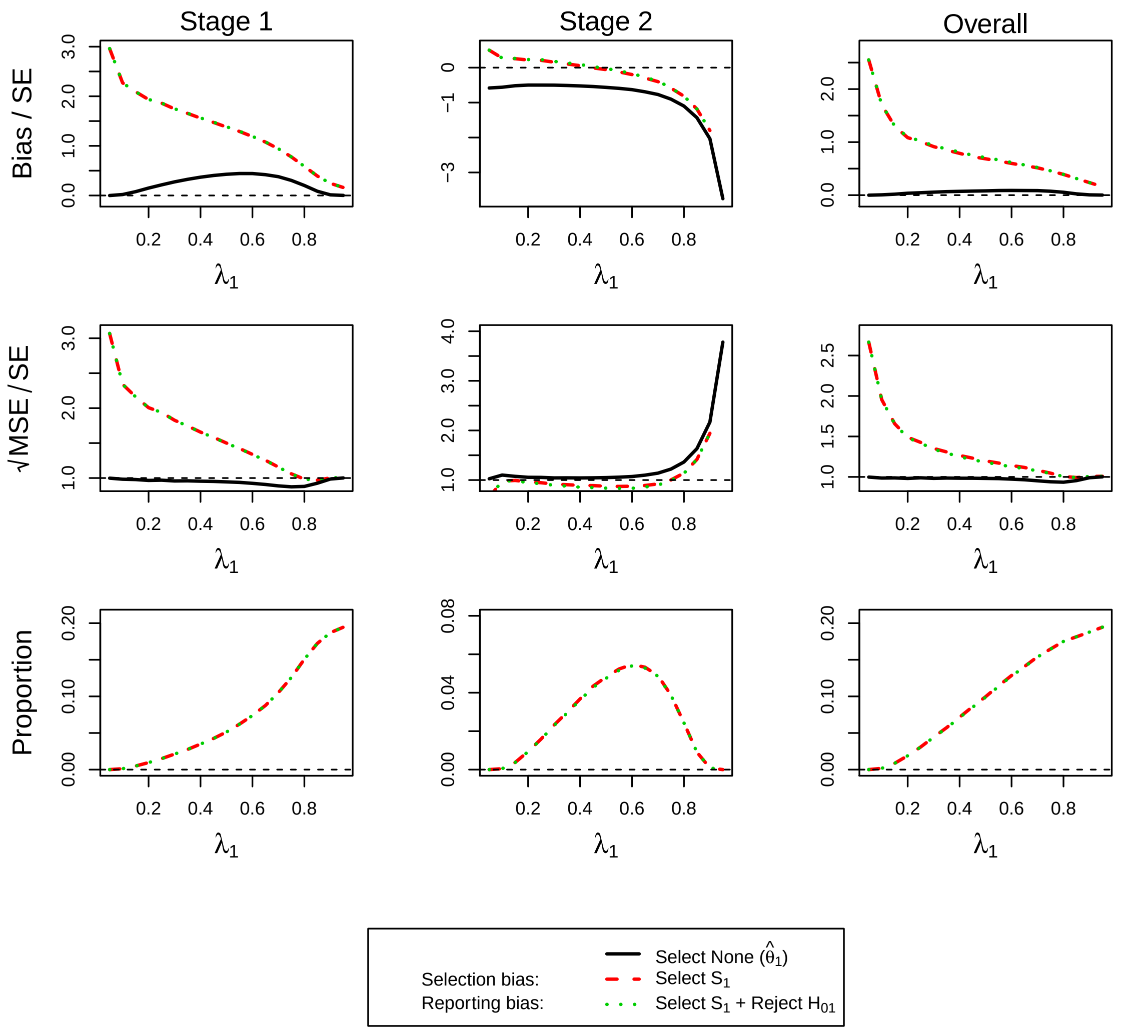

Figure S.5. (For **Design 3**, $\theta_1 = 0.5$ and $\theta_2 = 0.5$) standardized bias and MSE of $\hat{\theta}_1$ and simulation proportions for different circumstances at stopping stage 1, 2 and overall, against the prevalence of subpopulation 1, λ_1.

 Copyright © 2017 John Wiley & Sons, Ltd.

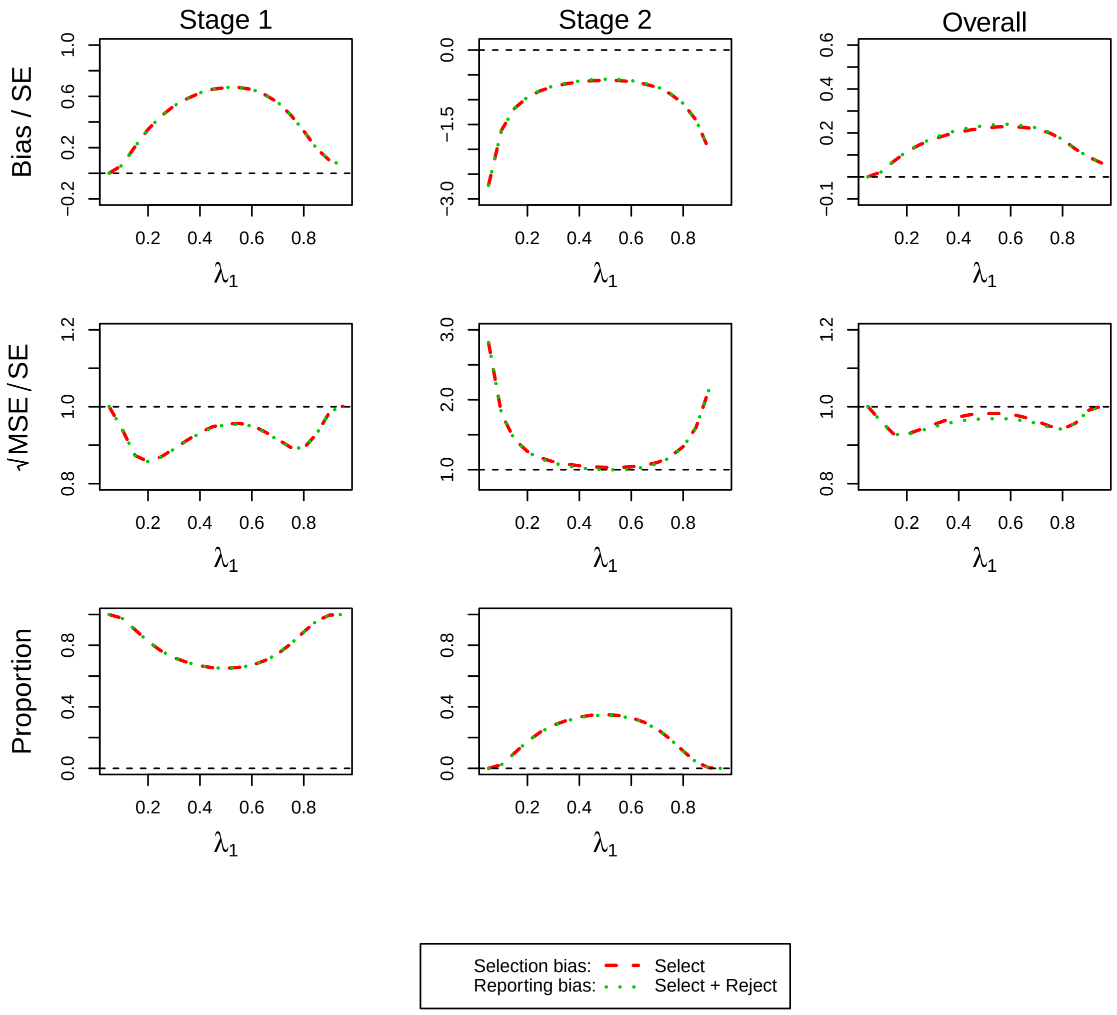

Figure S.6. (For **Design 3**, $\theta_1 = 0.5$ and $\theta_2 = 0.5$) family-wise bias and MSE and simulation proportions for different circumstances at stopping stage 1, 2 and overall, against the prevalence of subpopulation 1, λ_1.

Copyright © 2017 John Wiley & Sons, Ltd.

	Stop at Stage 1			Stop at Stage 2			Overall		
	Bias/SE	$\sqrt{\text{MSE}}$/SE	Prop.(%)	Bias/SE	$\sqrt{\text{MSE}}$/SE	Prop.(%)	Bias/SE	$\sqrt{\text{MSE}}$/SE	Prop.(%)
$\hat{\theta}_f$ (Select None)	0.33661	0.89132		-0.78128	1.14724		0.06013	0.95461	
$\hat{\theta}_f$ (Select F)	1.01674	1.21289	8.25	-0.39540	0.87995	3.82	0.56999	1.10756	12.07
$\hat{\theta}_f$ (Select F + Reject H_{0F})	1.01715	1.21252	8.25	-0.36132	0.82940	3.76	0.58602	1.09270	12.01
$\hat{\theta}_1$ (Select None)	0.30985	0.93105		-0.60669	1.09339		0.08317	0.97120	
$\hat{\theta}_1$ (Select S_1)	1.33691	1.47175	4.36	-0.07151	0.90106	2.68	0.80071	1.25449	7.04
$\hat{\theta}_1$ (Select S_1 + Reject H_{01})	1.33719	1.47162	4.36	-0.07034	0.89876	2.68	0.80142	1.25356	7.04
$\hat{\theta}_{1+2}$ (Select None)	0.41228	0.85587		-0.80790	1.16351		0.11049	0.93196	
$\hat{\theta}_{1+2}$ (Select S_{1+2})	0.50316	0.88596	62.66	-0.72961	1.10383	18.23	0.22527	0.93507	80.89
$\hat{\theta}_{1+2}$ (Select S_{1+2} + Reject $H_{0,1+2}$)	0.50329	0.88571	62.65	-0.72885	1.10232	18.23	0.22559	0.93453	80.88
Family-wise Select	0.60775	0.95572	75.27	-0.60671	1.04729	24.73	0.30738	0.97837	
Family-wise Select Select + Reject	0.60792	0.95546	75.26	-0.60138	1.03866	24.66	0.30944	0.97600	

Table S.4. For **Design 4**, $\theta_1 = 0.5$, $\theta_2 = 0.5$ and $\theta_3 = 0$) Standardized bias and standardized $\sqrt{\text{MSE}}$ of the MLEs where the prevalence rates of three subgroups are 1/3. In addition, Proportion (Prop.) stands for how often the corresponding circumstance occurs.

	Stop at Stage 1			Stop at Stage 2			Overall		
	Bias/SE	$\sqrt{\text{MSE}}$/SE	Prop.(%)	Bias/SE	$\sqrt{\text{MSE}}$/SE	Prop.(%)	Bias/SE	$\sqrt{\text{MSE}}$/SE	Prop.(%)
$\hat{\theta}_f$ (Select None)	0.13730	0.89448		-1.35869	1.55812		0.03899	0.93810	
$\hat{\theta}_f$ (Select F)	0.18764	0.89275	85.46	-1.32389	1.52367	5.51	0.09614	0.93040	90.97
$\hat{\theta}_f$ (Select F + Reject H_{0F})	0.18764	0.89275	85.46	-1.32360	1.52316	5.51	0.09617	0.93091	90.97
$\hat{\theta}_1$ (Select None)	0.08507	0.96518		-0.84141	1.24285		0.02419	0.98343	
$\hat{\theta}_1$ (Select S_1)	1.51904	1.66283	1.02	0.01030	0.91639	0.28	1.19387	1.50195	1.30
$\hat{\theta}_1$ (Select S_1 + Reject H_{01})	1.51904	1.66283	1.02	0.01254	0.91287	0.28	1.19453	1.50128	1.30
$\hat{\theta}_{1+2}$ (Select None)	0.11869	0.92729		-1.15762	1.42881		0.03481	0.96025	
$\hat{\theta}_{1+2}$ (Select S_{1+2})	0.79607	1.13413	6.94	-0.67677	1.05975	0.78	0.64655	1.12658	7.73
$\hat{\theta}_{1+2}$ (Select S_{1+2} + Reject $H_{0,1+2}$)	0.79607	1.13413	6.94	-0.67632	1.05875	0.78	0.64662	1.12648	7.73
Family-wise Select	0.24741	0.91910	93.42	-1.18971	1.44238	6.57	0.15296	0.95349	
Family-wise Select Select + Reject	0.24741	0.91910	33.77	-1.18936	1.44169	62.57	0.15300	0.95344	

Table S.5. For **Design 4**, $\theta_1 = 0.5$, $\theta_2 = 0.5$ and $\theta_3 = 0.5$) Standardized bias and standardized $\sqrt{\text{MSE}}$ of the MLEs where the prevalence rates of three subgroups are 1/3. In addition, Proportion (Prop.) stands for how often the corresponding circumstance occurs.

	Stop at Stage 1			Stop at Stage 2			Overall		
	Bias/SE	$\sqrt{\text{MSE}}$/SE	Prop.(%)	Bias/SE	$\sqrt{\text{MSE}}$/SE	Prop.(%)	Bias/SE	$\sqrt{\text{MSE}}$/SE	Prop.(%)
$\hat{\theta}_f$ (Select None)	-0.93679	1.17125		0.30256	0.95624		-0.08733	1.02388	
$\hat{\theta}_f$ (Select F)	-0.56853	0.81704	11.06	0.65069	1.05915	23.51	0.26069	0.98170	34.57
$\hat{\theta}_f$ (Select F + Reject H_{0F})	3.39993	3.40983	0.08	2.55316	2.57226	0.81	2.62926	2.64753	0.89
$\hat{\theta}_1$ (Select None)	-0.92616	1.16465		0.21979	0.95338		-0.14072	1.01984	
$\hat{\theta}_1$ (Select S_1)	-0.57368	0.81690	10.98	0.45633	1.02935	26.09	0.12259	0.96051	38.59
$\hat{\theta}_1$ (Select S_1 + Reject H_{01})	3.41824	3.43006	0.08	2.57227	2.59311	0.78	2.65305	2.67303	0.86
$\hat{\theta}_{1+2}$ (Select None)	-0.98107	1.20604		0.29132	0.95240		-0.10897	1.03219	
$\hat{\theta}_{1+2}$ (Select S_{1+2})	-0.54569	0.80728	7.90	0.59745	1.06136	18.94	0.26109	0.98660	26.84
$\hat{\theta}_{1+2}$ (Select S_{1+2} + Reject $H_{0,1+2}$)	3.41475	3.42472	0.07	2.57383	2.59524	0.67	2.65030	2.67067	0.74
Family-wise Select	-0.56484	0.81453	31.46	0.56200	1.04842	68.54	0.20750	0.97484	
Family-wise Select Select + Reject	3.41081	3.42141	0.23	2.56584	2.58623	2.25	2.64369	2.66317	

Table S.6. For **Design 4**, $\theta_1 = 0$, $\theta_2 = 0$ and $\theta_3 = 0$) Standardized bias and standardized $\sqrt{\text{MSE}}$ of the MLEs where the prevalence rates of three subgroups are 1/3. In addition, Proportion (Prop.) stands for how often the corresponding circumstance occurs.

Copyright © 2017 John Wiley & Sons, Ltd.

Statistics
in Medicine

References

1. Roweis S. *Gaussian Identities.* 1999. https://www.cs.nyu.edu/~roweis/notes/gaussid.pdf.

Copyright © 2017 John Wiley & Sons, Ltd.
